# Enantioselective Michael Addition of Aldehydes to Maleimides Organocatalyzed by a Chiral Primary Amine-Salicylamide

**DOI:** 10.3390/molecules23123299

**Published:** 2018-12-12

**Authors:** Alejandro Torregrosa-Chinillach, Adrien Moragues, Haritz Pérez-Furundarena, Rafael Chinchilla, Enrique Gómez-Bengoa, Gabriela Guillena

**Affiliations:** 1 Departamento de Química Orgánica, Facultad de Ciencias, and Instituto de Síntesis Orgánica (ISO), Uni­versidad de Alicante, Apdo. 99, 03080 Alicante, Spain; alextorregrosa96@gmail.com (A.T.-C.); adrien.moragues@gmail.com (A.M.); 2 Departamento de Química Orgánica I, Universidad del País Vasco, Apdo. 1072, 20080 San Sebastián, Spain; hperez017@ikasle.ehu.es

**Keywords:** organocatalysis, asymmetric synthesis, Michael addition, maleimides, aldehydes

## Abstract

A primary amine-salicylamide derived from chiral *trans*-cyclohexane-1,2-diamine was used as an organocatalyst for the enantioselective conjugate addition of aldehydes, mainly α,α-disubstituted to *N*-substituted maleimides. The reaction was performed in toluene as a solvent at room temperature. The corresponding enantioenriched adducts were obtained with high yields and enantioselectivities up to 94%. Theoretical calculations were used to justify the stereoinduction.

## 1. Introduction

Maleimides have been successfully used as building blocks in many asymmetric organocatalytic transformations for the preparation of compounds of interest [1]. Among the compounds that can be prepared by the organocatalytic functionalization of maleimides, succinimides are one of the most important since the succinimide moiety is present in natural products and some clinical drug candidates [2,3,4,5,6]. Moreover, succinimides can be transformed into other interesting compounds, such as γ-lactams [7,8], which are important in the treatment of HIV [9,10], epilepsy [11,12], and other neurological disorders [13].

The most direct way of preparing enantioenriched substituted succinimides is by the organocatalytic enantioselective conjugate addition of carbon nucleophiles to maleimides [1]. These carbon nucleophiles can be generated by the α-deprotonation of pro-nucleophiles bearing acidic α-hydrogens, such as 1,3-dicarbonyl compounds, by means of chiral organocatalysts that contain both a tertiary amine suitable to deprotonate the pro-nucleophile as well as an acidic moiety [1]. The subsequent formation of a close transition state, which involves the coordination of the maleimide to the catalyst by means of a hydrogen bond and the enolate generated after deprotonation by the tertiary amine, leads to an efficient enantioselective process.

**Figure 1:**
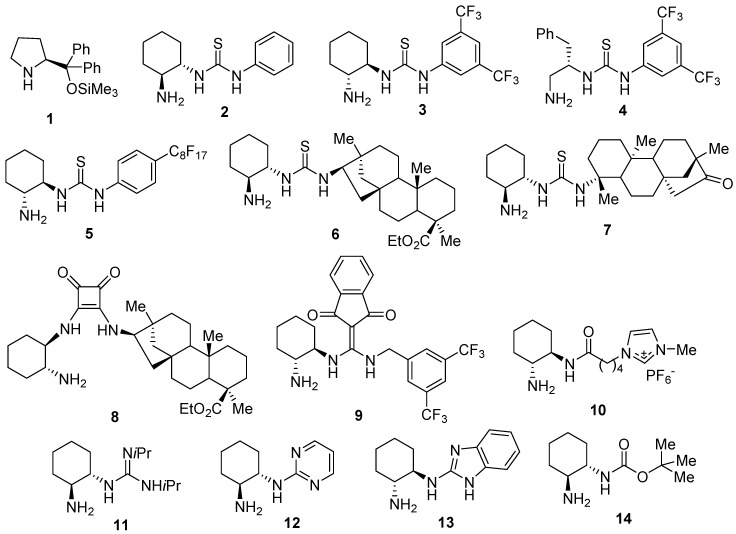
Chiral organocatalysts employed in the enantioselective Michael addition of aldehydes to maleimides.

However, when aldehydes are used as pro-nucleophiles, the α-deprotonation process becomes much more difficult. The corresponding conjugate addition can be obtained using primary amine-bearing organocatalysts that are amenable to create transition states after the formation of a transient enamine with the pro-nucleophile. Thus, the first organocatalytic Michael addition of aliphatic aldehydes to *N*-aryl-maleimides used α,α-phenylprolinol silyl ether **1** as an organocatalyst, affording much lower enantioselectivities when α,α-disubstituted aldehydes were employed [14] (Figure 1). This type of diarylated prolinol has also been employed as an organocatalyst working in ionic liquids, although only with linear aldehydes [15].

Different bifunctional primary amine-bearing organocatalysts are suitable to an additional coordination to the carbonyl group of the maleimide via hydrogen bonds, and are frequently prepared from chiral *trans*-cyclohexa-1,2-diamines. Taking into consideration this enamine-forming approach, these organocatalysts have been applied to the enantioselective Michael addition of aldehydes to maleimides, giving good enantioselectivities of the corresponding succinimides (Figure 1). Thus, the primary amine-thioureas **2** [16], **3** [17,18], **4** [19], and **5** [20], the beyerane-containing thiourea **6** [21], the isosteviol-derived thiourea **7** [22], the squaramide **8** [23], and even the calixarene-based thioureas [24,25], have been successfully employed. In addition, primary amino acid derivatives [26,27] and dipeptides have also been used [28], as well as a combination of Cinchona alkaloid-derived thioureas and primary amino acids [29] or Cinchona alkaloid-derived primary amines [30]. Other bifunctional primary amine-containing compounds used as organocatalysts in this transformation have been the diaminomethyleneindenedione derivative **9** [31], the ionic liquid-supported amide **10** [32], the guanidine **11** [33,34], the 2-aminopyrimidine **12** [35], the 2-benzimidazole **13** [36], the carbamate **14** [37,38,39], and the polymer-supported sulfonamides [40]. Even the simple non-derivatized chiral *trans*-cyclohexa-1,2-diamines have been used as organocatalysts [41].

**Figure 2:**
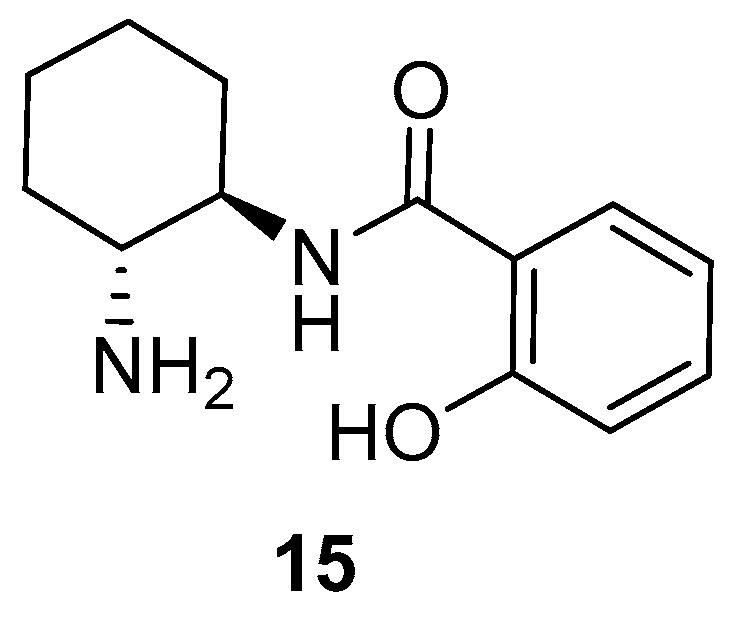
The organocatalyst employed in this study.

Recently, our research group used a chiral primary amine-monosalicylamide from *trans*-cyclohexa-1,2-diamine (**15** and *ent*-**15**, Figure 2) as an organocatalyst for the enantioselective Michael addition of α,α-disubstituted aldehydes to β-nitroalkenes, obtaining the corresponding γ-nitroaldehydes in an excellent chemical yield and with enantioselectivities up to 95% [42]. This study discusses the results that were obtained when this organocatalyst was employed in the enantioselective Michael addition of aldehydes to *N*-substituted maleimides. The results are explained by theoretical calculations.

## 2. Results and Discussion

The primary amine-salicylamide **15** was prepared as reported by the monoamidation of (1*R*,2*R*)-cyclohexane-1,2-diamine with phenyl salicylate in refluxing propan-2-ol [42]. The search for the most appropriate reaction conditions (Table 1) was carried out using the model Michael addition reaction of isobutyraldehyde (**16a**) to *N*-phenylmaleimide (**17a**). Thus, the reaction organocatalyzed by **15** (10 mol%) in toluene as a solvent at room temperature afforded the corresponding substituted succinimide (*R*)-**18aa** almost quantitatively and in an excellent 94% *ee* after 2 days reaction time (Table 1, entry 1). The (*R*) absolute configuration of the final adduct was determined by comparing the elution order of the corresponding enantiomers in chiral HPLC with those in the literature [38]. The use of chlorinated solvents, such as dichloromethane or chloroform, afforded good yields but lower enantioselectivities for (*R*)-**18aa** (Table 1, entries 2 and 3). 

When a 2/1 *v*/*v* mixture of DMF/H_2_O was used as a solvent, the enantiomeric (*S*)-**18aa** was obtained in 79% *ee* (Table 1, entry 4). This inversion in the enantioselectivity of the process was also observed when the related primary amine-monocarbamate **14** was employed as an organocatalyst, and attributed to a loss of the bifunctional character of the catalyst due to the competitive hydrogen-bond formation with water [38].

We also assayed the influence of the addition of some acid or basic additives. Thus, when benzoic acid (10 mol%) was added to the reaction mixture in toluene, (*R*)-**18aa** was obtained in only 77% *ee* (Table 1, entry 5), an enantioselectivity that rose up to 85% when LiCl was employed as a non-protic acid (Table 1, entry 6). The addition of an organic base, such as 4-*N*,*N*-dimethylaminopyridine (DMAP), gave similar *ee* than before, but in a much lower chemical yield (Table 1, entry 7).

**Figure 3:**
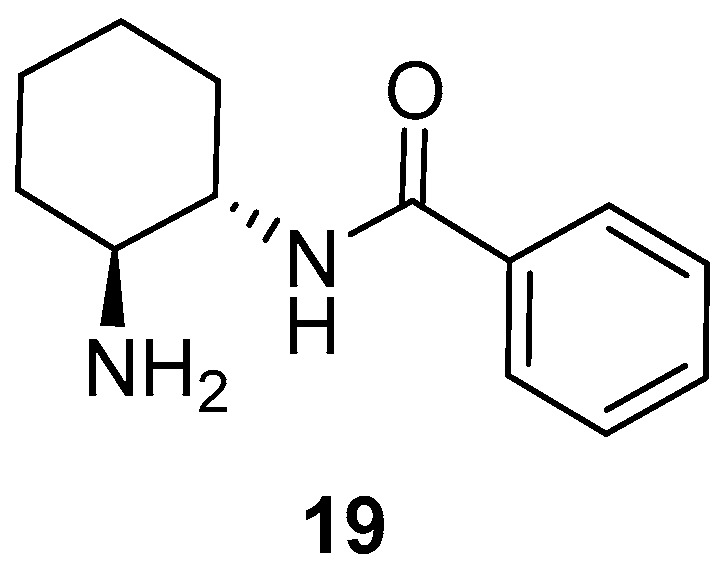
The OH-free organocatalyst employed in this study.

We were curious to determine if the presence of the phenolic OH on the organocatalyst was the determinant for achieving a high enantioselectivity. Thus, as the organocatalyst, we employed the primary amine-containing benzamide **19**, obtained by the reaction of (1*S*,2*S*)-cyclohexane-1,2-diamine with phenyl benzoate under similar conditions as **15** (Figure 3) [38]. However, under the above optimal reaction conditions, this organocatalyst **19** gave rise to adduct (*S*)-**18aa** in a lower 87% *ee* (Table 1, entry 8). Therefore, the presence of the phenolic OH in organocatalyst **15** had an influence on the enantioselectivity of the reaction. It is interesting to note that the use of a “related” monocarbamate organocatalyst **14** only gave a 67% *ee* for (*S*)-**18aa** when using toluene as a solvent [38].

Next, we extended the enantioselective Michael addition reaction to other aldehydes **16** and maleimides **17** under the most favorable reaction conditions [**15** (10 mol%), toluene, rt], the results of which are summarized in Table 2. The absolute configuration of the known succinimides **18** was assigned in accordance with the elution order of the enantiomers in chiral HPLC when compared to the literature (see Experimental Section).

Thus, when **16a** reacted with *N*-arylmaleimides **17b** and **17c** bearing electron-donating groups on the phenyl ring, such as 4-methyl and 4-methoxy, the corresponding Michael adducts (*R*)-**18ab** and (*R*). **18ac** were obtained with similar enantioselectivities (88% and 89%, respectively) (Table 2, entries 2 and 3). In addition, when the *N*-arylmaleimide **17d** bearing a chloro group at the *para*-position was used, adduct (*R*)-**18ad** was obtained in an 88% *ee* (Table 2, entry 4). The presence of electron-withdrawing groups on the phenyl ring gave rise to a lower enantioselection. Thus, when the *N*-substituted maleimide **17e** bearing a 4-acetyl group was employed with isobutyraldehyde, succinimide (*R*)-**18ae** was obtained in only a 13% *ee* (Table 2, entry 5). This value increased to 70% when maleimide **17f**, bearing a 4-nitro group, was used as an electrophile (Table 2, entry 6). In addition, an *N*-alkylated maleimide, such as *N*-methylmaleimide (**17g**), was also used as an electrophile, affording succinimide (*R*)-**18ag** with a 82% *ee* (Table 2, entry 7). However, when the simple maleimide (**17h**) was employed, the final adduct (*R*)-**18ah** was isolated in a lower enantioselectivity (56%) (Table 2, entry 8).

We also explored the conjugate addition reaction of other α,α-disubstituted aldehydes with maleimide **17a**. Thus, when cyclopentanecarbaldehyde (**16b**) was used, the corresponding Michael adduct (*R*)-**18ba** was isolated in an excellent yield and with an enantioselectivity of 82% (Table 2, entry 9). The use of cyclohexanecarbaldehyde (**16c**) as a pro-nucleophile gave only a 36% *ee* (Table 2, entry 10). In addition, when propionaldehyde (**16d**) was employed as a pro-nucleophile, a 1/1.2 mixture of diastereomers was isolated and (2*S*,3*R*)-**18da** and (2*R*,3*R*)-**18da** were obtained in 79% and 89% *ee*, respectively (Table 2, entry 11).

**Figure 4:**
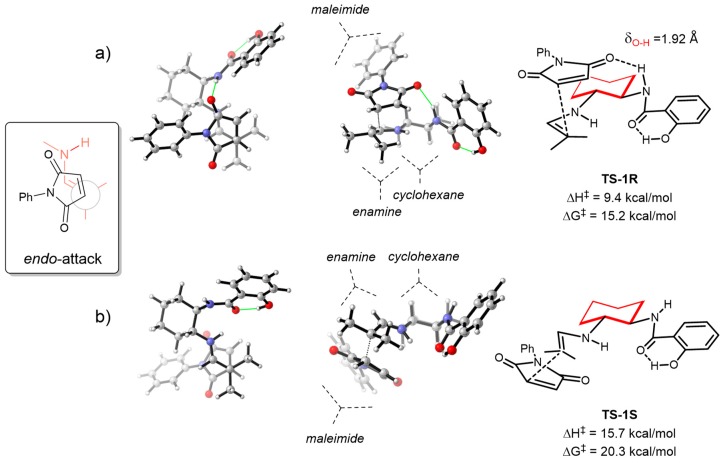
Transition states for the formation of (*R*)-**18aa** (**a**) and the (*S*)-**18aa** (**b**) catalyzed by **15**. 3D-Newman projections (left-figures), side views (middle-figures) and 2-D representations are shown.

To acquire further insight into the origin of the enantioselectivity, we carried out theoretical calculations on the reaction between isobutyraldehyde **16a** and maleimide **17a** in the presence of organocatalyst **15**. According to our previous computational results on a related process [38], the reaction proceeds by the formation of an enamine, followed by an attack to the electrophilic maleimide substrate through an *endo* transition state (Figure 4). We also considered the possible *exo* approach, but the higher activation energies were sufficient enough to discard its participation in the process. In this situation, the two faces of the enamine were clearly differentiated. If the approach of the maleimide occurred through the upper face (from our view), as it did in **TS-1R**, an efficient H-bond between the C=O group of maleimide and the NH group of the catalyst was formed. This activated the electrophile and induced a low activation barrier (15.2 kcal/mol) for the formation of the *R* product. If the approach of the two reactants was occurring from the other face, as it was in **TS-1S**, the carbonyl and NH moieties were far enough to avoid the formation of any effective H-bond, and the activation energy could not be lowered, resulting in a value of 20.3 kcal/mol. Thus, the high energy difference of the two transition states nicely explained the formation of the experimental major (*R*)-**18aa** isomer. 

**Figure 5:**
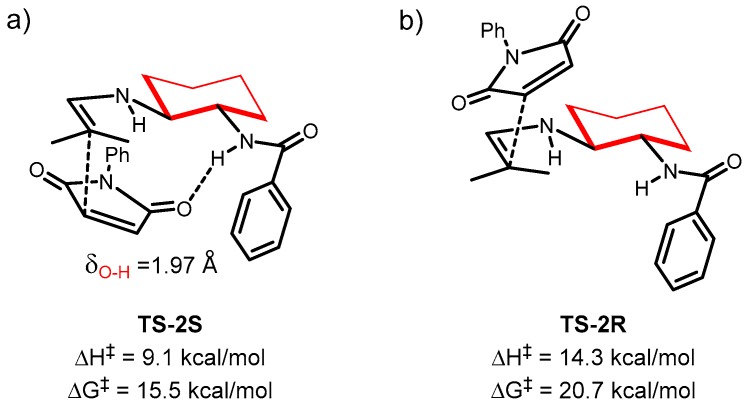
Transition states for the formation of (*S*)-**18aa** (**a**) and (*R*)-**18aa** (**b**) catalyzed by **19**.

Meanwhile, the diastereomeric transition states for the reaction organocatalyzed by **19** were also computed. As mentioned before, catalyst **19** lacked the phenolic OH group, and showed similar reactivity and moderately lower enantioselectivity under similar conditions as catalyst **15** (Table 1, entries 1 and 8). Interestingly, the optimal transition states for **19** were located (**TS-2S** and **TS-2R**, Figure 5) and showed quite similar activation parameters as catalyst **15**, although with enough differences to explain a moderate decrease in the enantioselectivity. For example, the Gibbs Free activation barriers leading to the two enantiomers were almost equivalent (15.2 vs 15.5 kcal/mol and 20.3 vs 20.7 kcal/mol), but the activation enthalpy difference between **TS-1R** and **TS-1S** was 6.3 kcal/mol, while the same value for **TS-2S** vs **TS-2R** was 5.2 kcal/mol. Thus, the presence of the OH induced a slight increase in the enthalpy gap between the two faces of the maleimide. Also, the critical H-bonding distances differed slightly, being shorter for **TS-1R** than for **TS-2S** (compare δ_O-H_ in Figure 4 and Figure 5). Thus, these data indicated that the H-bonding activation of the maleimide was optimal when the phenolic OH was present, lowering the enthalpy barrier in **TS-1R** the effects helped to increase the enantioselectivity observed when organocatalyst **15** was employed, rigidifying the NH-CO-Ar benzamide system, which reduced the conformational variability of catalyst **19**.

## 3. Experimental Section

### 3.1. General Information 

All of the reagents and solvents employed were of the best grade available and were used without further purification. The ^1^H spectra were recorded at room temperature on a Bruker Oxford AV300 at 300 MHz, using TMS as the internal standard. Absolute configuration for adducts **18** was determined according to the order of elution of their enantiomers in chiral HPLC. Reference racemic samples of adducts **18** were obtained by performing the conjugate addition reaction using 4-methylbenzylamine (20 mol%) as an organocatalyst in toluene as a solvent at room temperature.

### 3.2. General Procedure for the Asymmetric Conjugate Addition Reaction

A solution of **15** (0.02 mmol, 4,7 mg) and the maleimide **17** (0.2 mmol) in toluene (0.5 mL) was added to the aldehyde **16** (0.4 mmol), and the mixture was stirred at rt for 48 h (TLC). The reaction was quenched with HCl 2 N (10 mL) and the mixture was extracted with AcOEt (3 × 10 mL). The organic phase was washed with saturated NaHCO_3_ (10 mL) and brine (10 mL), dried over MgSO_4_, filtered, and the solvent was then evaporated (15 Torr) to get the crude product, which was purified by silica gel chromatography (*n*-hexane/AcOEt gradients). Adducts **18** were identified by the comparison of their ^1^H-NMR data with those of the literature. Their enantiomeric excesses were determined by chiral HPLC using the conditions described in each case.

*2-(2,5-Dioxo-1-phenylpyrrolidin-3-yl)-2-methylpropanal* (**18aa**) [38]. White solid (48 mg, 98%); ^1^H-NMR (CDCl_3_): δ*_H_* = 9.52 (s, 1H), 7.48 (m, 2H), 7.40 (m, 1H), 7.28 (m, 2H), 3.16 (dd, *J* = 9.5, 5.5 Hz, 1H), 2.99 (dd, *J* = 18.2, 9.5 Hz, 1H), 2.63 (dd, *J* = 18.2, 5.5 Hz, 1H), 1.34 (s, 3H), 1.29 (s, 3H) ppm; HPLC: Chiralcel OD-H, λ = 210 nm, *n*-hexane/2-propanol, 80:20, 1.0 mL/min, t_r_ (*minor*) = 23.5 min, t_r_ (*major*) = 27.2 min.

*2-(2,5-Dioxo-1-(p-tolyl)pyrrolidin-3-yl)-2-methylpropanal* (**18ab**) [23]. White solid (45 mg, 87%); ^1^H-NMR (CDCl_3_): δ*_H_* 9.52 (s, 1H), 7.27 and 7.15 (AA’BB’ system, 4H), 3.15 (dd, *J* = 9.5, 5.4 Hz, 1H), 2.96 (dd, *J* = 18.3, 9.5 Hz, 1H), 2.60 (dd, *J* = 18.2, 5.4 Hz, 1H), 2.37 (s, 3H), 1.31 (s, 3H), 1.28 (s, 3H) ppm; HPLC: Chiralcel OD-H, λ = 210 nm, *n*-hexane/2-propanol, 80:20, 1.0 mL/min, t_r_ (*minor*) = 18.9 min, t_r_ (*major*) = 20.8 min. 

*2-(1-(4-Methoxyphenyl)-2,5-dioxopyrrolidin-3-yl)-2-methylpropanal* (**18ac**) [17]. White solid (42 mg, 77%); ^1^H-NMR (CDCl_3_): δ*_H_* = 9.52 (s, 1H), 7.19 and 6.98 (AA’BB’ system, 4H), 3.82 (s, 3H), 3.14 (dd, *J* = 9.5, 5.4 Hz, 1H), 2.97 (dd, *J* = 18.2, 9.4 Hz, 1H), 2.60 (dd, *J* = 18.2, 5.3 Hz, 1H), 1.33 (s, 3H), 1.28 (s, 3H) ppm; HPLC: Chiralpak AS-H, λ = 210 nm, *n*-hexane/2-propanol, 80:20, 1.0 mL/min): t_r_ (*minor*) = 41.6 min, t_r_ (*major*) = 44.4 min.

*2-(1-(4-Chlorophenyl)-2,5-dioxopyrrolidin-3-yl)-2-methylpropanal* (**18ad**) [38]. White solid (51 mg, 92%); ^1^H-NMR (CDCl_3_): δ*_H_* = 9.49 (s, 1H), 7.45 and 7.25 (AA’BB’ system, 4H), 3.12 (dd, *J* = 9.5, 5.4 Hz, 1H), 2.98 (dd, *J* = 18.1, 9.5 Hz, 1H), 2.62 (dd, *J* = 18.1, 5.5 Hz, 1H), 1.37 (s, 3H), 1.29 (s, 3H) ppm; HPLC: Chiralcel OD-H, λ = 210 nm, *n*-hexane/2-propanol, 80:20, 1.0 mL/min, t_r_ (*minor*) = 21.8 min, t_r_ (*major*) = 37.0 min.

*2-(1-(4-Acetylphenyl)-2,5-dioxopyrrolidin-3-yl)-2-methylpropanal* (**18ae**) [38]. White solid (42 mg, 73%); ^1^H-NMR (CDCl_3_): δ*_H_* = 9.50 (s, 1 H), 8.06 and 7.45 (AA’BB’ system, 4H), 3.14 (dd, *J* = 9.5, 5.5 Hz, 1H), 3.01 (dd, *J* = 18.1, 9.6 Hz, 1H), 2.66 (dd, *J* = 18.2, 5.6 Hz, 1 H), 2.62 (s, 3 H), 1.38 (s, 3 H), 1.31 (s, 3 H) ppm; HPLC: Chiralpak AS-H, λ = 210 nm, *n*-hexane/2-propanol, 80:20, 1.0 mL/min): t_r_ (*minor*) = 31.6 min, t_r_ (*major*) = 41.5 min.

*2-Methyl-2-(1-(4-nitrophenyl)-2,5-dioxopyrrolidin-3-yl)propanal* (**18af**) [24]. White solid (45 mg, 78%); ^1^H-NMR (CDCl_3_): δ*_H_* = 9.47 (s, 1H), 8.35 and 7.60 (AA’BB’ system, 4H), 3.11 (dd, *J* = 9.6, 5.2 Hz, 1H), 3.02 (dd, *J* = 17.7, 9.6 Hz, 1H), 2.66 (dd, *J* = 17.7, 5.2 Hz, 1H), 1.43 (s, 3H), 1.32 (s, 3H) ppm; HPLC: Chiralpak AD-H, λ = 210 nm, *n*-hexane/2-propanol, 65:35, 1.0 mL/min, t_r_ (*minor*) = 12.1 min, t_r_ (*major*) = 26.6 min.

*2-Methyl-2-(1-methyl-2,5-dioxopyrrolidin-3-yl)propanal* (**18ag**) [38]. Colorless oil (33 mg, 91%); ^1^H-NMR (CDCl_3_): δ*_H_* = 9.51 (s, 1H), 3.03 (dd, *J* = 9.3, 5.4 Hz, 1H), 2.98 (s, 3H), 2,83 (dd, *J =* 18.2, 9.3 Hz), 2.41 (dd, *J* = 18.2, 5.3 Hz, 1H), 1.23 (s, 3H), 1.21 (s, 3H) ppm; HPLC: Chiralpak AS-H, λ = 210 nm, *n*-hexane/2-propanol, 80:20, 1.0 mL/min, t_r_ (*major*) = 12.8 min, t_r_ (*minor*) = 14.2 min.

*2-(2,5-Dioxopyrrolidin-3-yl)-2-methylpropanal* (**18ah**) [38]. White solid (24 mg, 71%); ^1^H-NMR (CDCl_3_): δ*_H_* = 9.49 (s, 1 H), 7.95 (br. s, 1 H), 3.09 (dd, *J* = 9.4, 5.8 Hz, 1H), 2.86 (dd, *J* = 18.4, 9.4 Hz, 1H), 2.51 (dd, *J* = 18.4, 5.8 Hz, 1H), 1.26 (s, 3H), 1.25 (s, 3H) ppm; HPLC: Chiralpak AD-H, λ = 210 nm, *n*-hexane/2-propanol, 80:20, 1.0 mL/min, t_r_ (*major*) = 17.3 min, t_r_ (*minor*) = 23.2 min.

*1-(2,5-Dioxo-1-phenylpyrrolidin-3-yl)cyclopentane-1-carbaldehyde* (**18ba**) [38]. White solid (50 mg, 93%); ^1^H-NMR (CDCl_3_): δ*_H_* = 9.39 (s, 1H), 7.50–7.23 (m, 5H), 3.01 (m, 2H), 2.58 (dd, *J* = 17.6, 9.6 Hz, 1H), 2.30 (dd, *J* = 17.6, 5.2 Hz, 1H), 2.07-1.97 (m, 2H), 1.90-1.73 (m, 5H) ppm; HPLC: Chiralcel OD-H, λ = 210 nm, *n*-hexane/2-propanol, 80:20, 0.5 mL/min, t_r_ (*minor*) = 21.8 min, t_r_ (*major*) = 27.3 min.

*1-(2,5-Dioxo-1-phenylpyrrolidin-3-yl)cyclohexane-1-carbaldehyde* (**18ca**) [38]. White solid (48 mg, 84%); ^1^H-NMR (CDCl_3_): δ*_H_* = 9.55 (s, 1H), 7.50–7.23 (m, 5H), 3.22 (dd, *J* = 9.4, 5.9 Hz, 1H), 2.88 (dd, *J* = 18.2, 9.4 Hz, 1H), 2.68 (dd, *J* = 18.1, 5.9 Hz, 1H), 1.99-1.45 (m, 10H) ppm; HPLC: Chiralcel OD-H, λ = 210 nm, *n*-hexane/2-propanol, 80:20, 0.9 mL/min, t_r_ (*minor*) = 20.8 min, t_r_ (*major*) = 25.7 min.

*2-(2,5-Dioxo-1-phenylpyrrolidin-3-yl)propanal* (**18da**) [38]. Yellow oil (45 mg, 98%); Mixture of diastereomers (ratio: 1/1.2*, ^1^H-NMR). ^1^H-NMR (CDCl_3_): δ*_H_* = 9.63/9.54* (s, 1H), 7.43–7.19 (m, 10H), 3.33/3.20 (m, 1H), 3.10/3.00 (m, 1H), 3.02/2.97 (dd, *J* = 18.5, 9.6 Hz, 1H), 2.61/2.55 (dd, *J* = 17.9, 5.5 Hz, 1H), 1.30/1.25 (d, *J* = 7.8 Hz, 3H) ppm; HPLC: Chiralpak AD-H, λ = 210 nm, *n*-hexane/2-propanol, 80:20, 0.8 mL/min, diastereomer 1: t_r_ (*minor*) = 17.5 min, t_r_ (*major*) = 25.9 min, diastereomer 2: t_r_ (*minor*)= 18.9 min, t_r_ (*major*) = 19.7 min.

### 3.3. Computational Methods.

All reported structures were optimized at Density Functional Theory level using the B3LYP [43,44,45] functional as implemented in Gaussian 09 [46]. Optimizations were carried out with the 6-31G(d,p) basis set. The stationary points were characterized by frequency calculations to verify that they had the right number of imaginary frequencies. The reported energy values correspond to Gibbs Free energies, including single point refinements at M06-2X/6-311 + G(d,p) [47] level of theory in a solvent model (IEFPCM, toluene) [48,49,50] on the previously optimized structures (computed structures in the Supplementary Materials).

## 4. Conclusions

We conclude that a primary amine-salicylamides, prepared by a simple monoamidation of an enantiomerically pure *trans*-cyclohexane-1,2-diamine, acts as an efficient organocatalyst in the enantioselective conjugate addition of aldehydes to maleimides, leading to enantio­merically enriched succinimides. Good yields and enantioselectivities can be achieved working in toluene as a solvent at room temperature. Theoretical calculations suggest that the phenolic OH present in catalyst **15** helps to preorganize the system, inducing a more effective H-bonding of the benzamide NH towards the activation of the maleimide. The activation can only be effective in one of the faces of the maleimide (**TS-1R**), leading to a high degree of enantioselectivity with **15**.

## Figures and Tables

**Table 1:**
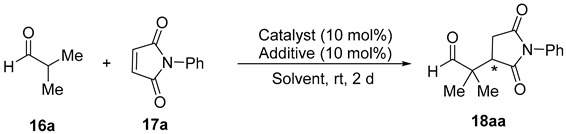
Screening and optimization of the reaction conditions for the model enantioselective Michael addition.

Entry	Catalyst	Additive	Solvent	Yield (%) ^a^	*ee* (%) ^b^
1	**15**	-	PhMe	98	94 (*R*)
2	**15**	-	CH_2_Cl_2_	96	74 (*R*)
3	**15**	-	CHCl_3_	98	75 (*R*)
4	**15**	-	DMF/H_2_O ^c^	91	79 (*S*)
5	**15**	PhCO_2_H	PhMe	94	77 (*R*)
6	**15**	LiCl	PhMe	88	85 (*R*)
7	**15**	DMAP	PhMe	45	83 (*R*)
8	**19**	-	PhMe	98	87 (*S*)

^a^ Isolated yield after flash chromatography. ^b^ Enantioselectivities and absolute stereochemistry determined by chiral HPLC. ^c^ 2/1 *v*/*v*.

**Table 2:**
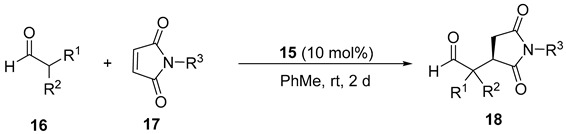
Enantioselective Michael addition of aldehydes to maleimides organocatalyzed by **15**.

Entry	Aldehyde		Maleimide		Michael Adduct		
	R^1^, R^2^	No.	R^3^	No.	No.	Yield (%) ^a^	*ee*^b^ (%) ^b^
1	Me,Me	**16a**	Ph	**17a**	(*R*)-**18aa**	98	94
2	Me,Me	**16a**	4-MeC_6_H_4_	**17b**	(*R*)-**18ab**	87	88
3	Me,Me	**16a**	4-MeOC_6_H_4_	**17c**	(*R*)-**18ac**	77	89
4	Me,Me	**16a**	4-ClC_6_H_4_	**17d**	(*R*)-**18ad**	92	88
5	Me,Me	**16a**	4-AcC_6_H_4_	**17e**	(*R*)-**18ae**	73	13
6	Me,Me	**16a**	4-O_2_NC_6_H_4_	**17f**	(*R*)-**18af**	78	70
7	Me,Me	**16a**	Me	**17g**	(*R*)-**18ag**	91	82
8	Me,Me	**16a**	H	**17h**	(*R*)-**18ah**	71	56
9	-(CH_2_)_4_-	**16b**	Ph	**17a**	(*R*)-**18ba**	93	82
10	-(CH_2_)_5_-	**16c**	Ph	**17a**	(*R*)-**18ca**	84	36
11	Me,H	**16d**	Ph	**17a**	(2*S*,3*R*)-,(2*R*,3*R*)-**18da** ^c^	98 ^d^	79,89 ^e^

^a^ Isolated yield after flash chromatography. ^b^ Enantioselectivities determined by chiral HPLC. Absolute configuration assigned by the order of elution of the enantiomers in chiral HPLC (See Experimental Section). ^c^ 1/1.2 diastereomer ratio (^1^H-NMR). ^d^ Combined yield. ^e^ Enantioselectivities referred to (2*S*,3*R*) and (2*R*,3*R*), respectively.

## Supplementary Materials

The following are available online at http://www.mdpi.com/1420-3049/23/12/3299/s1, NMR spectra, HPLC chromatograms and cartesian coordinates of the computed structures.
