# Supplementary material for: Enantioselective Michael Addition of Aldehydes to Maleimides Organocatalyzed by a Chiral Primary Amine-Salicylamide
Source: Molecules. 2018 Dec 12;23(12):3299. doi: 10.3390/molecules23123299 (PMC6320823; doi:10.3390/molecules23123299)
Supplement: Supplementary file 1 [file molecules-23-03299-s001.pdf]

Supplementary Materials:

Enantioselective Michael addition of aldehydes to maleimides organocatalyzed by a chiral primary amine-salicylamide

Alejandro Torregrosa-Chinillach, Adrien Moragues, Haritz Pérez-Furundarena, Rafael Chinchilla,* Enrique Gómez-Bengoa,* Gabriela Guillena*

^{1}H NMR spectra and HPLC chromatograms

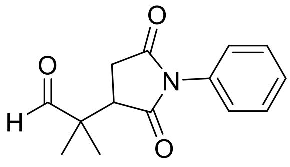

18aa

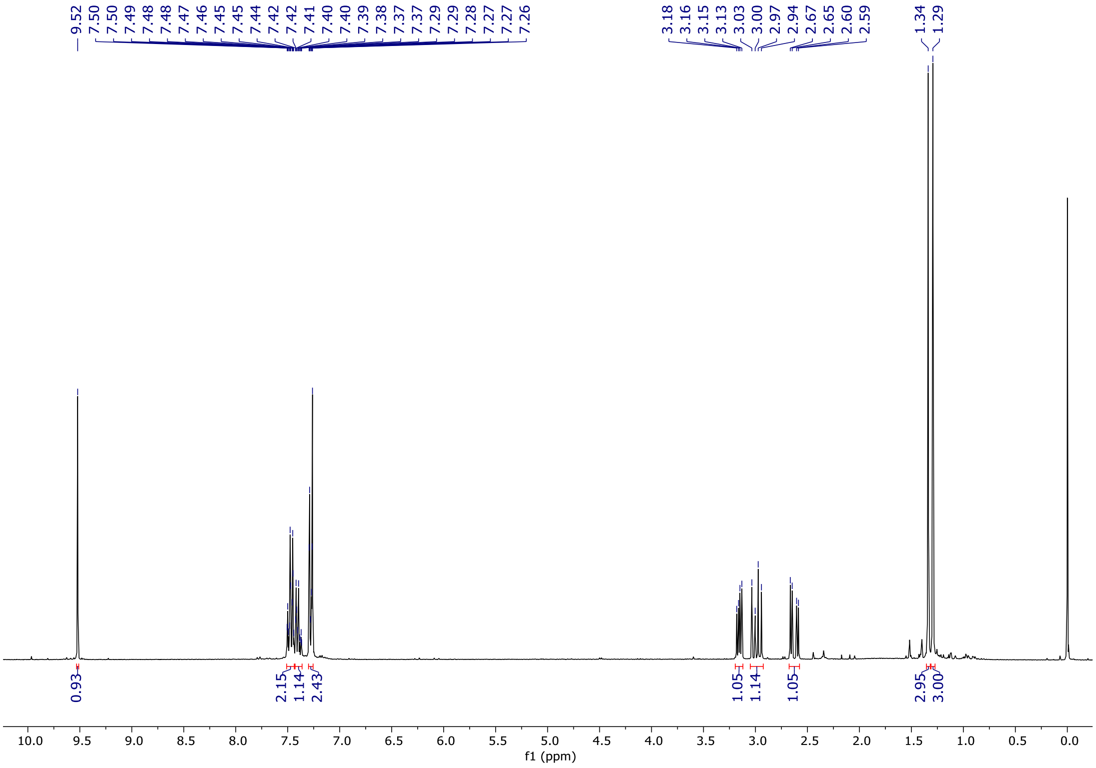

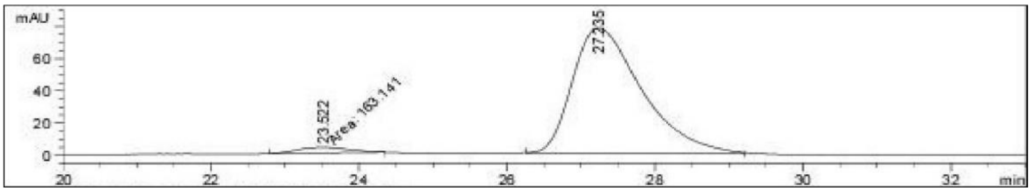

Peak #	RetTime [min]	Type	Width [min]	Area [mAU*s]	Height [mAU]	Area %
1	23.522	MM	0.78	163.14	3.48	3.14
2	27.235	BB	0.92	5028.85	77.73	96.86

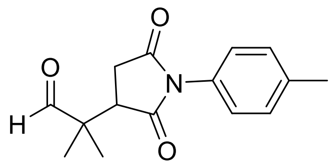

18ab

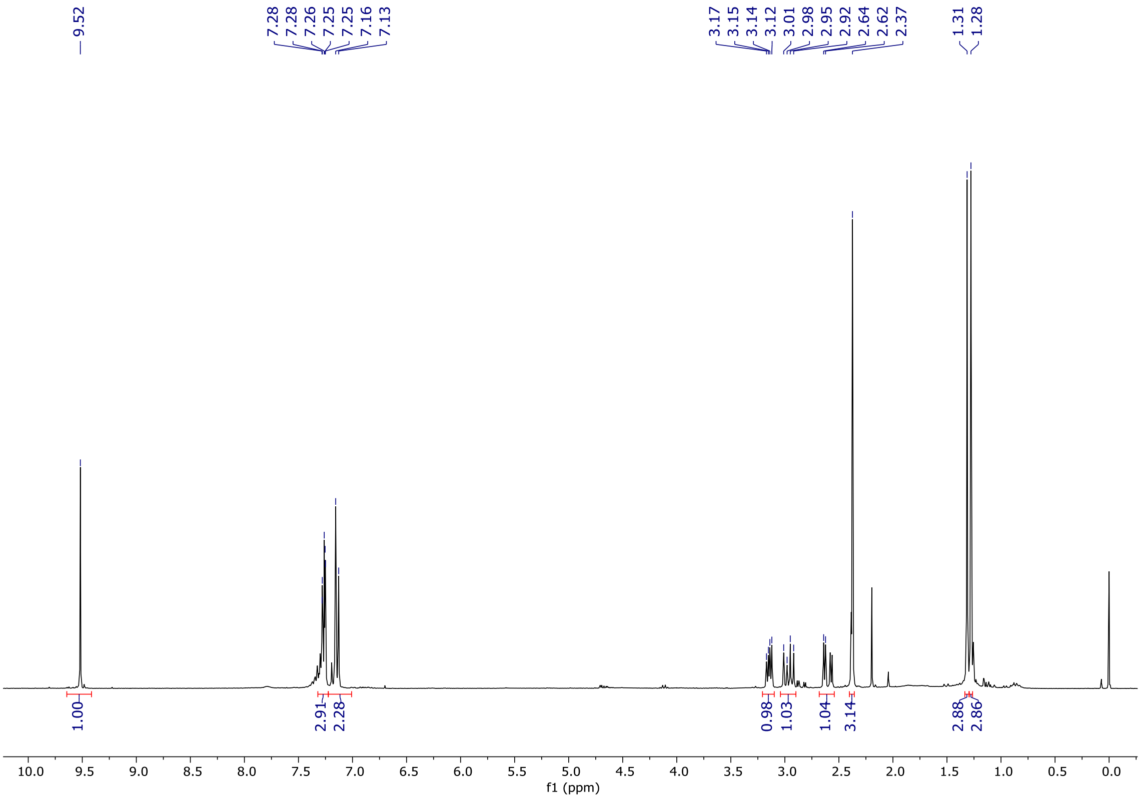

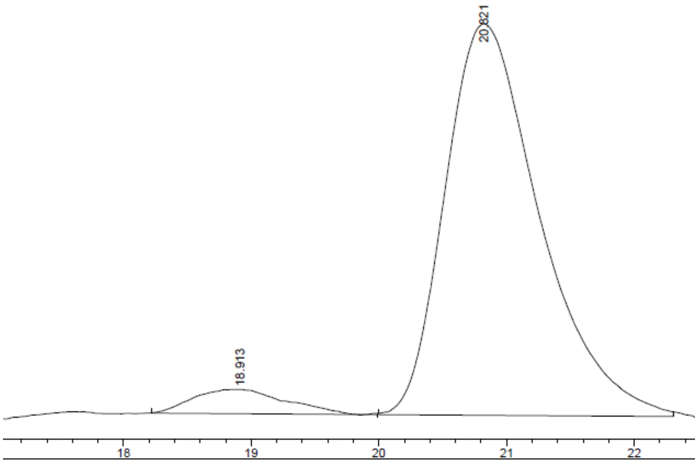

Peak #	RetTime [min]	Type	Width [min]	Area [mAU*s]	Height [mAU]	Area %
1	18.913	BB	0.6072	196.51817	3.86466	5.8223
2	20.821	BB	0.7592	3178.73779	61.68907	94.1777

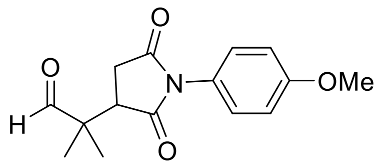

18ac

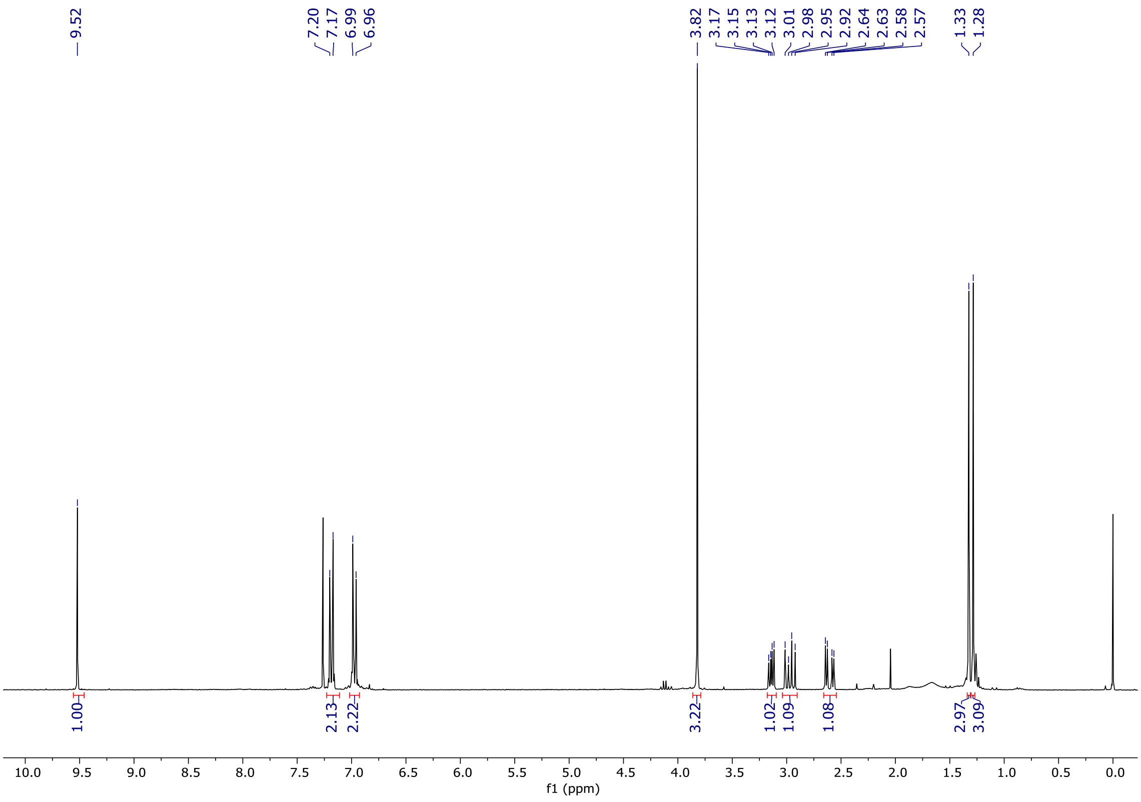

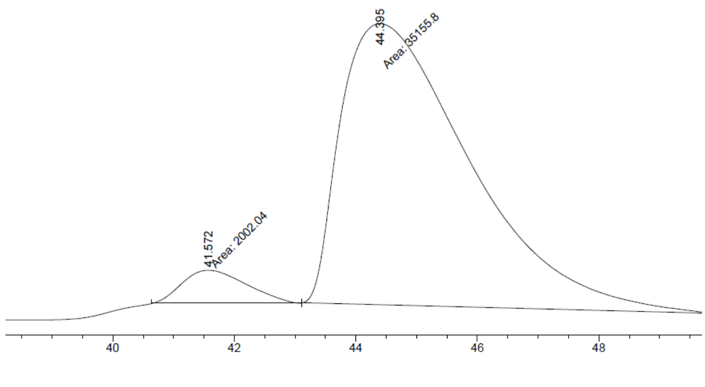

Peak #	RetTime [min]	Type	Width [min]	Area [mAU*s]	Height [mAU]	Area %
1	41.572	MM	1.2081	2002.03589	27.62063	5.3879
2	44.395	MM	2.4573	3.51558e4	238.44348	94.6121

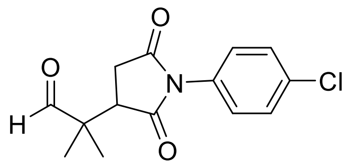

18ad

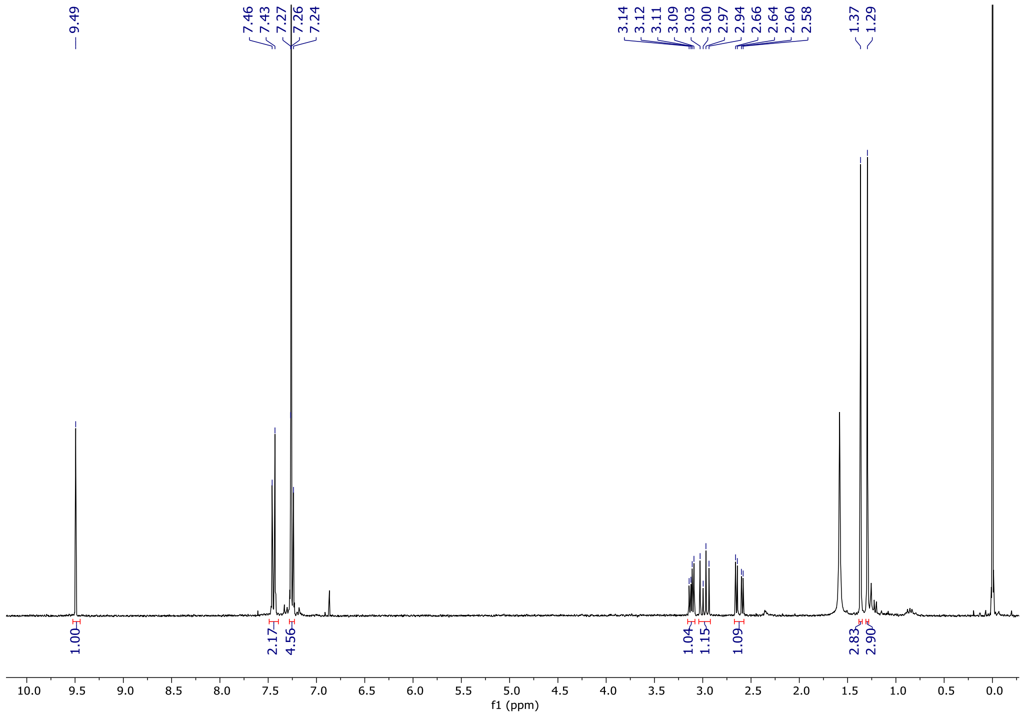

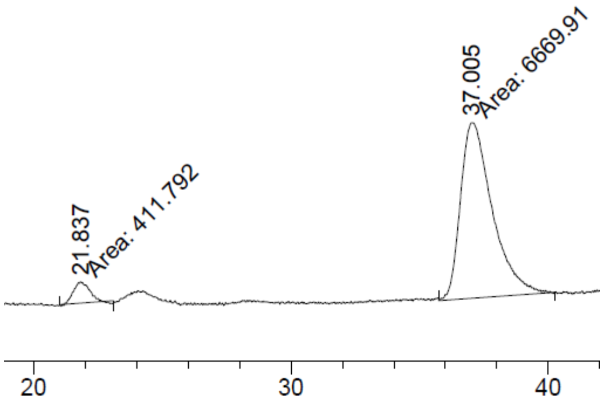

Peak #	RetTime [min]	Type	Width [min]	Area [mAU*s]	Height [mAU]	Area %
1	21.837	MM	0.7213	411.79233	9.51546	5.8149
2	37.005	MM	1.4320	6669.90967	77.62900	94.1851

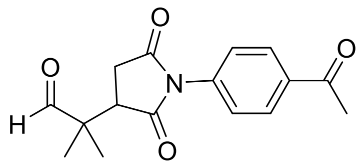

18ae

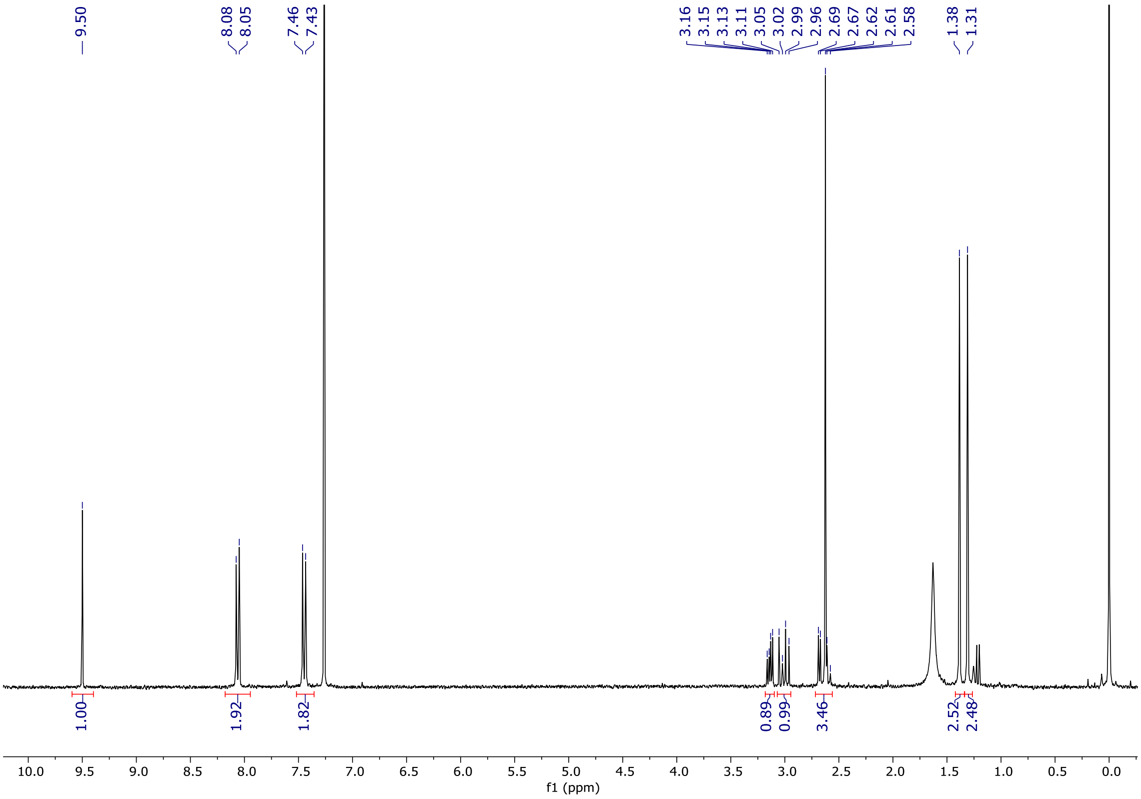

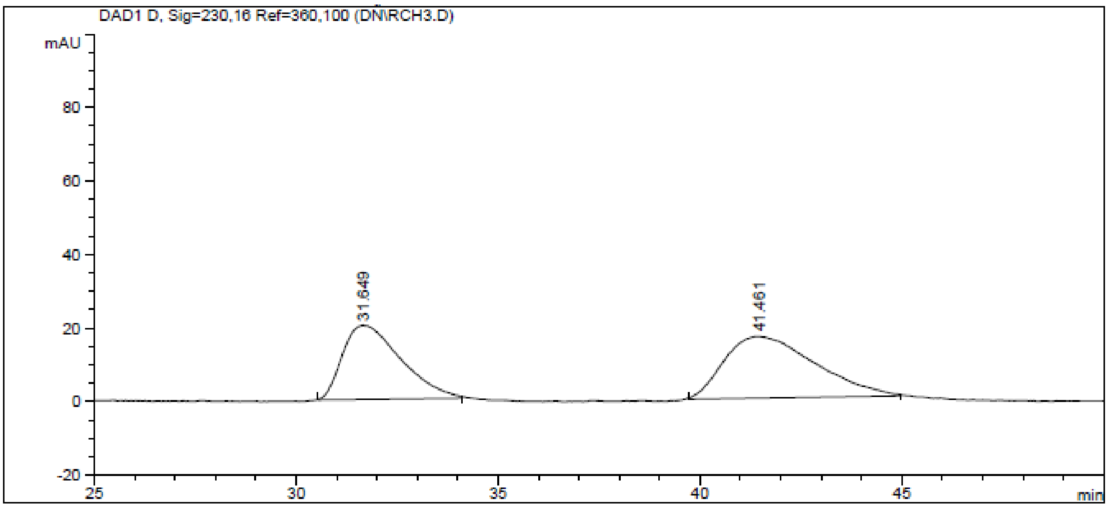

Peak #	RetTime [min]	Type	Width [min]	Area [mAU*s]	Height [mAU]	Area %
1	31.649	BB	1.1917	2029.12036	20.23937	43.4919
2	41.461	BB	1.8556	2636.38892	16.79266	56.5081

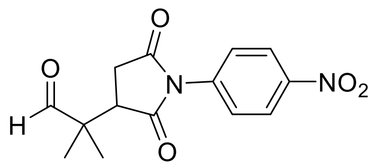

18af

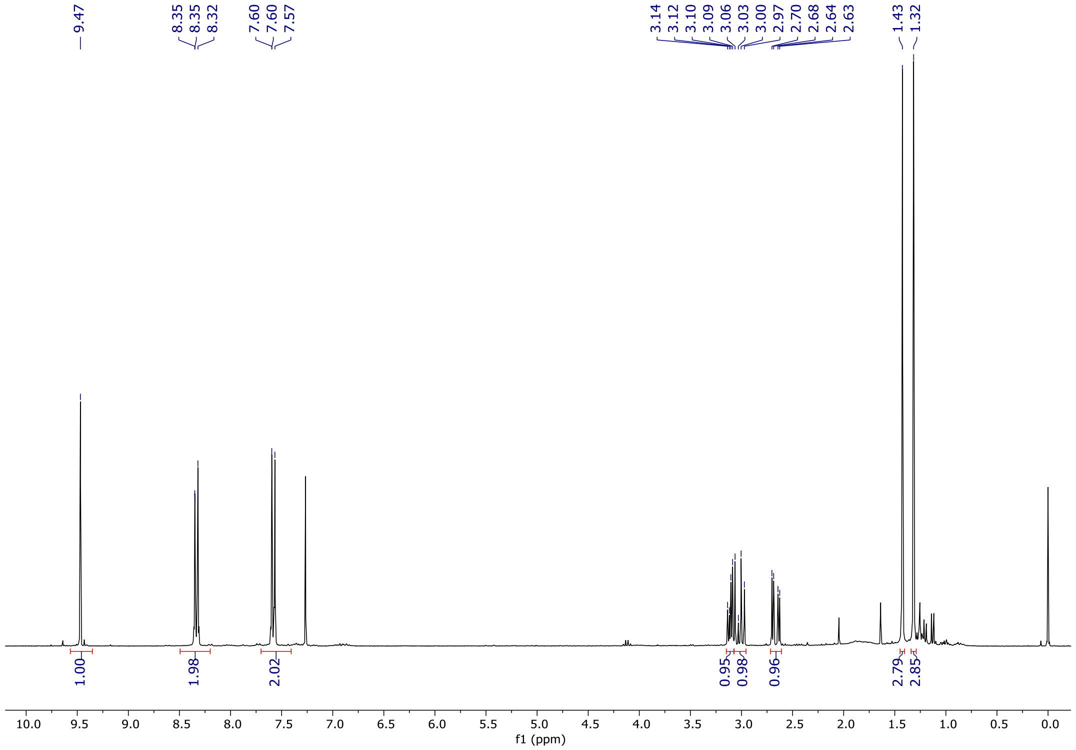

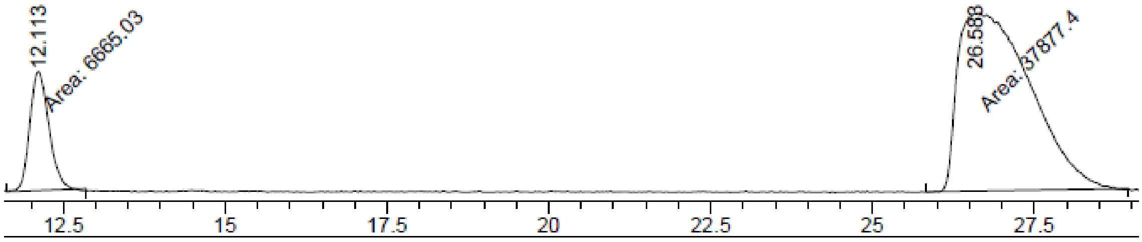

Peak #	RetTime [min]	Type	Width [min]	Area [mAU*s]	Height [mAU]	Area %
1	12.113	MM	0.3436	6665.03223	323.28546	14.9633
2	26.583	MM	1.3037	3.78774e4	484.23560	85.0367

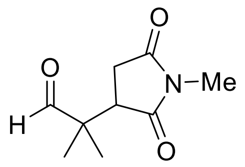

18ag

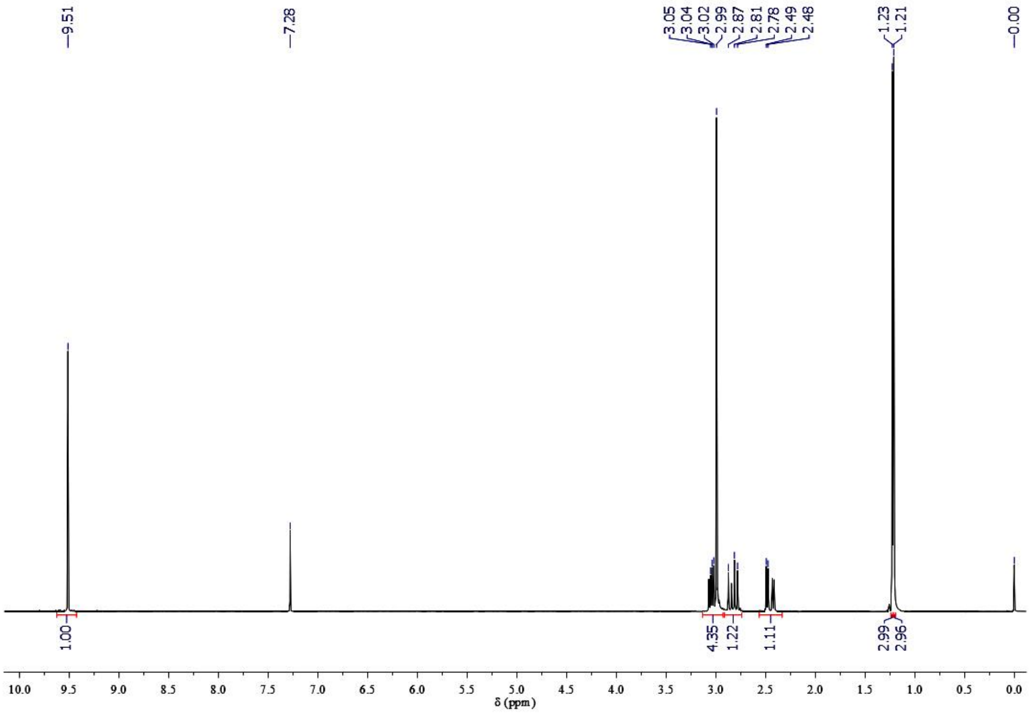

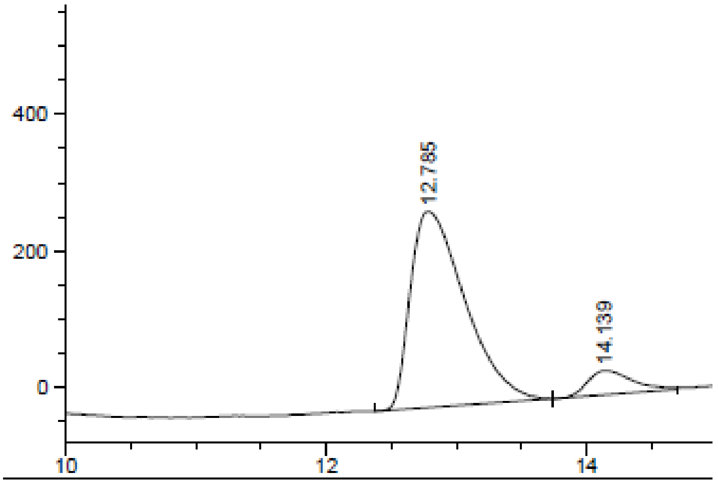

Peak #	RetTime [min]	Type	Width [min]	Area [mAU*s]	Height [mAU]	Area %
1	12.785	PP	0.4493	8396.49512	287.05466	90.7502
2	14.139	BV	0.3608	855.82037	35.55899	9.2498

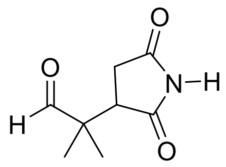

18ah

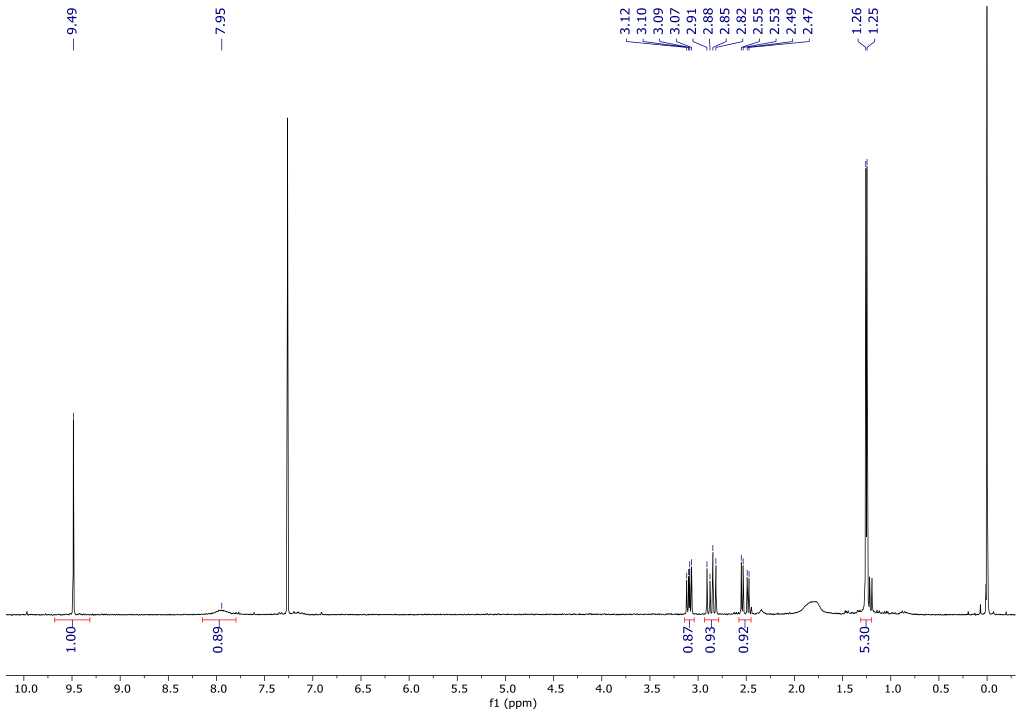

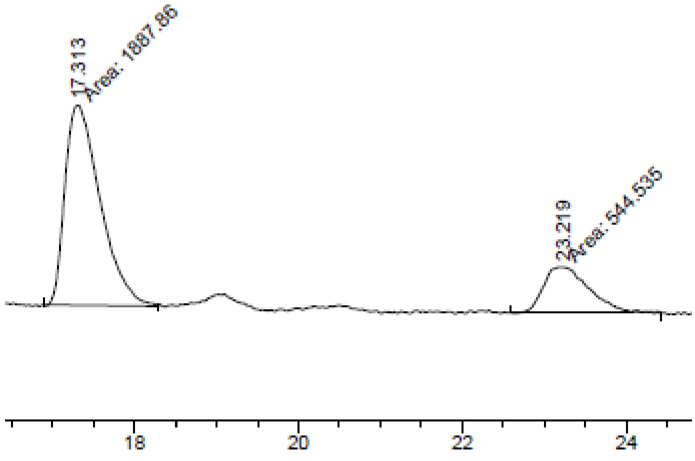

Peak #	RetTime [min]	Type	Width [min]	Area [mAU*s]	Height [mAU]	Area %
1	17.313	MM	0.4993	1887.86304	63.02269	77.6132
2	23.219	MM	0.6300	544.53534	14.40677	22.3868

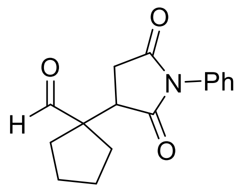

18ba

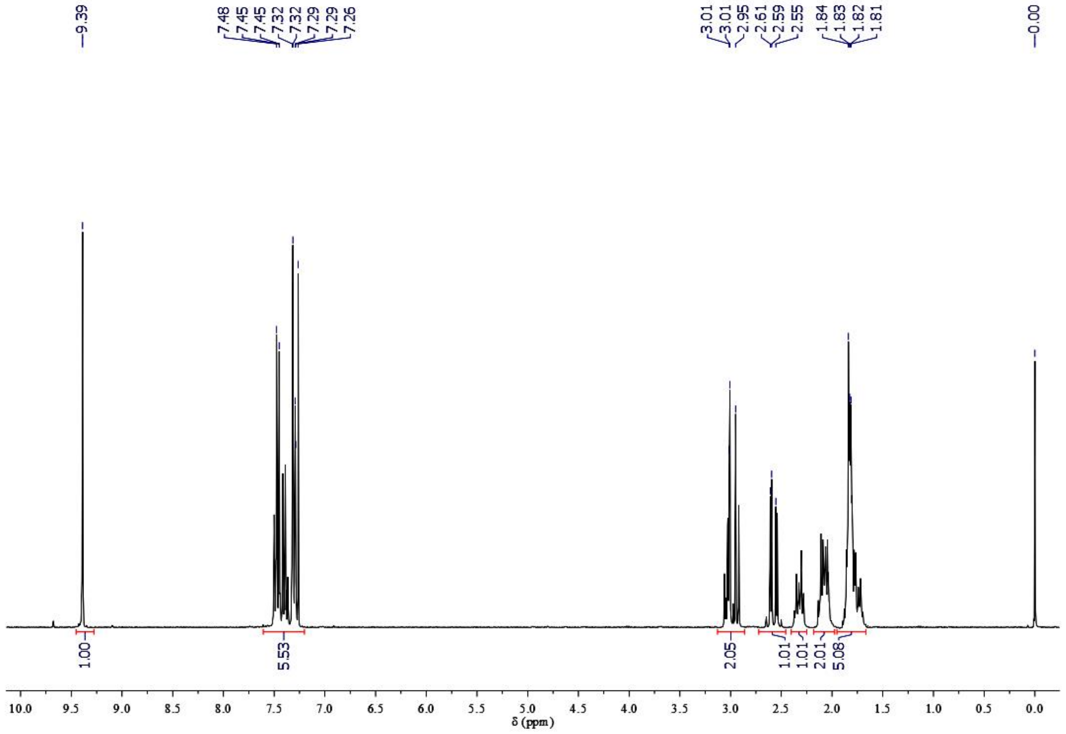

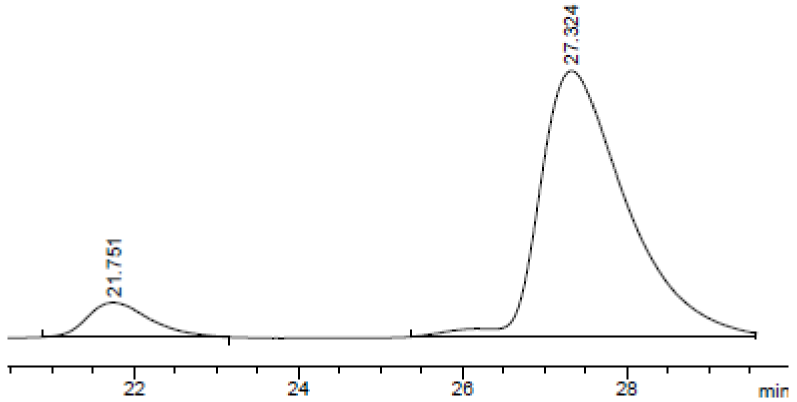

Peak #	RetTime [min]	Type	Width [min]	Area [mAU*s]	Height [mAU]	Area %
1	21.751	BB	0.6458	1327.12219	24.50738	8.8007
2	27.324	BBA	1.0545	1.37525e4	186.90102	91.1993

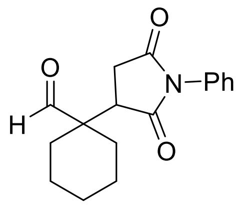

18ca

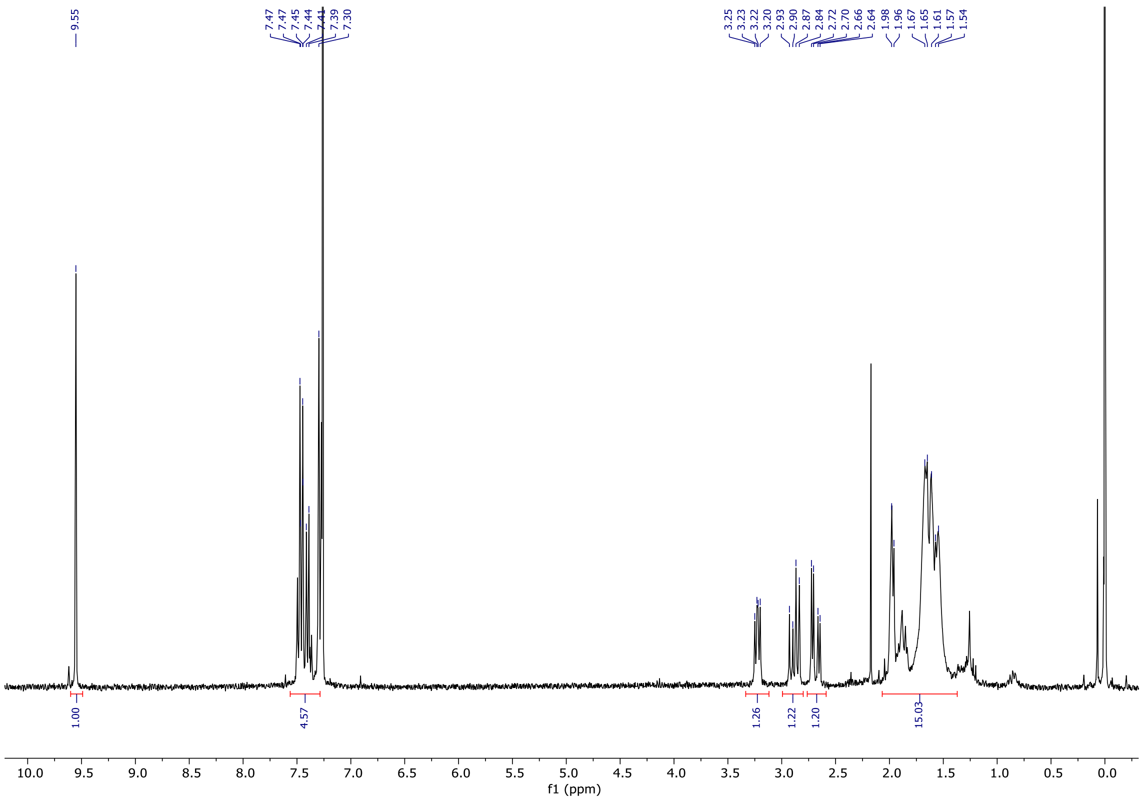

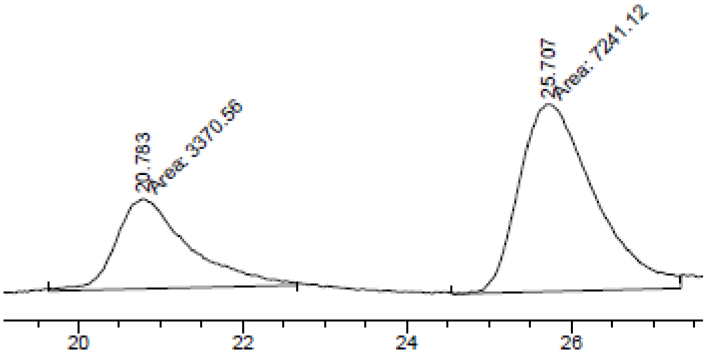

Peak #	RetTime [min]	Type	Width [min]	Area [mAU*s]	Height [mAU]	Area %
1	20.783	MM	1.0307	3370.56055	54.50221	31.7628
2	25.707	MM	1.0623	7241.11621	113.60856	68.2372

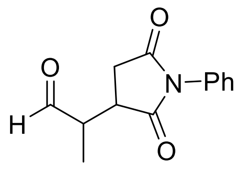

18da

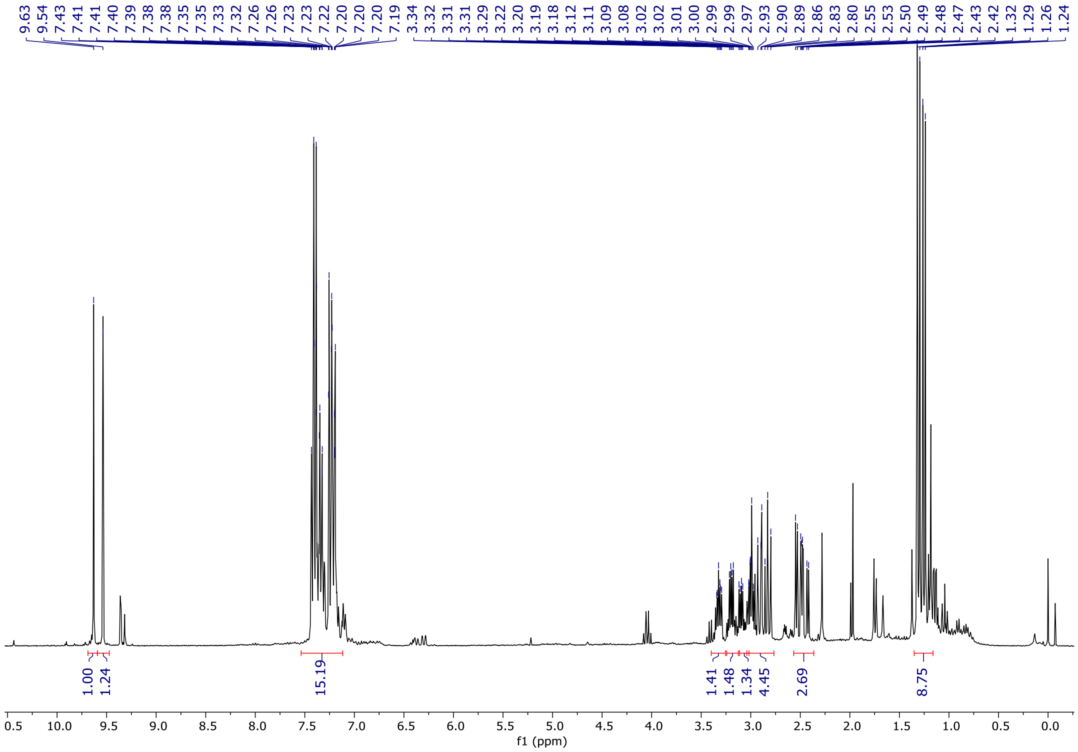

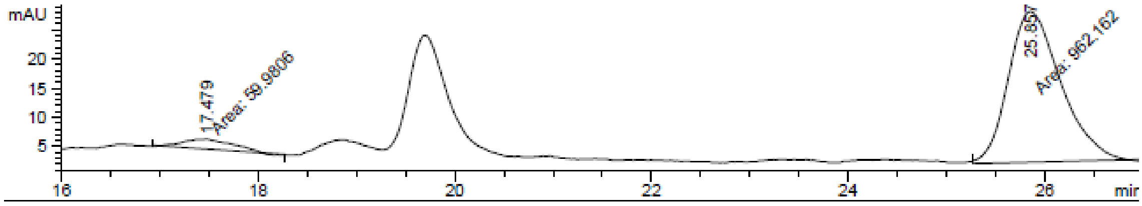

Peak #	RetTime [min]	Type	Width [min]	Area [mAU*s]	Height [mAU]	Area %
1	17.479	MM	0.5795	59.98059	1.72519	5.8681
2	25.857	MM	0.6208	962.16193	25.83209	94.1319

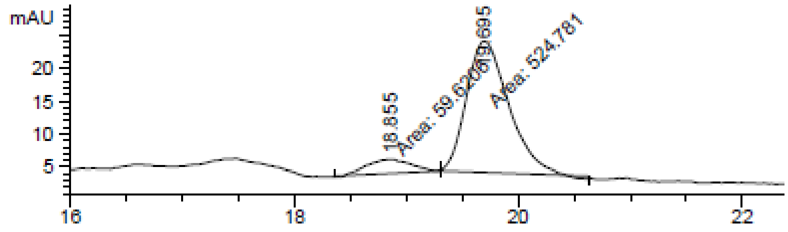

Peak #	RetTime [min]	Type	Width [min]	Area [mAU*s]	Height [mAU]	Area %
1	18.855	MM	0.4686	59.62057	2.12067	10.2020
2	19.695	MM	0.4402	524.78149	19.86934	89.7980

Cartesian coordinates of the computed structures

Maleimide **17a**
E(SCRF) (M06-2X/def2tzvpp): -590,377082 Hartrees
Correction to Enthalpy: 0.160012
Correction to Gibbs Free Energy: 0.112975
Standard orientation:

Center Number	Atomic Number	Atomic Type	Coordinates (Angstroms) X	Y	Z
1	6	0	2.994112	0.652767	-0.138004
2	6	0	-1.362907	1.074336	0.564592
3	6	0	-2.756733	-1.071343	-0.553363
4	6	0	-1.362905	-1.074353	-0.564592
5	6	0	-0.666283	-0.000014	0.000013
6	6	0	2.994128	-0.652736	0.137941
7	6	0	-3.458752	-0.000006	-0.000008
8	6	0	1.575728	-1.129335	0.236030
9	6	0	-2.756735	1.071331	0.553348
10	6	0	1.575701	1.129356	-0.235967
11	7	0	0.761961	-0.000003	0.000014
12	8	0	1.195607	-2.256339	0.468559
13	8	0	1.195544	2.256339	-0.468541
14	1	0	3.826734	1.328209	-0.282364
15	1	0	-3.293213	1.910254	0.986219
16	1	0	-4.544477	-0.000004	-0.000015
17	1	0	-3.293211	-1.910261	-0.986246
18	1	0	-0.818827	-1.906676	-0.992196
19	1	0	-0.818830	1.906664	0.992189
20	1	0	3.826768	-1.328172	0.282226

Enamine of catalyst **15**
E(SCRF) (M06-2X/def2tzvpp): -922,208385 Hartrees
Correction to Enthalpy: 0.411283
Correction to Gibbs Free Energy: 0.338581
Standard orientation:

Center Number	Atomic Number	Atomic Type	Coordinates (Angstroms) X	Y	Z
1	6	0	4.280546	-1.907720	0.198236
2	6	0	4.836793	-0.563285	-0.292479
3	6	0	3.943761	0.606649	0.145119
4	1	0	4.902695	-0.578242	-1.389505
5	1	0	5.858162	-0.417148	0.077695
6	6	0	2.483963	0.427240	-0.310864
7	1	0	3.960677	0.697008	1.240898
8	6	0	1.937796	-0.928750	0.217145
9	6	0	2.820584	-2.103319	-0.234859
10	1	0	2.774686	-2.188061	-1.331898
11	1	0	2.415887	-3.036822	0.171975
12	1	0	4.336702	-1.946314	1.295180
13	1	0	4.893129	-2.736199	-0.173820
14	7	0	0.547836	-1.159956	-0.165703
15	1	0	1.936967	-0.883616	1.310734
16	1	0	4.316285	1.556626	-0.249900

17	6	0	-0.503376	-0.806674	0.617015
18	1	0	0.363461	-1.420696	-1.122636
19	8	0	-0.314615	-0.262681	1.728736
20	6	0	-1.876238	-1.083926	0.123684
21	6	0	-2.969493	-0.492904	0.810243
22	6	0	-4.277162	-0.690852	0.334508
23	6	0	-2.145950	-1.902489	-0.989905
24	6	0	-3.439236	-2.107162	-1.444690
25	6	0	-4.505934	-1.485156	-0.778447
26	1	0	-5.090228	-0.216133	0.871606
27	1	0	-1.330348	-2.415195	-1.492776
28	1	0	-3.622968	-2.748229	-2.300807
29	1	0	-5.523301	-1.635816	-1.128285
30	8	0	-2.808901	0.262919	1.908681
31	1	0	-1.840554	0.217718	2.131020
32	1	0	2.474474	0.388736	-1.409799
33	1	0	-1.719463	2.660677	1.158991
34	1	0	-0.043823	2.953187	1.634930
35	6	0	-0.797983	3.186745	0.877405
36	1	0	-2.264368	3.008553	-1.544376
37	6	0	-0.361373	2.836395	-0.523850
38	6	0	-1.228664	3.367758	-1.635892
39	1	0	-1.009459	4.261638	0.957281
40	1	0	1.317311	1.479681	1.033016
41	7	0	1.665066	1.566612	0.084545
42	6	0	0.727585	2.097928	-0.808485
43	1	0	-0.860749	3.067075	-2.621340
44	1	0	-1.281595	4.465601	-1.620125
45	1	0	0.980532	1.914608	-1.852602

Enamine of catalyst **19**
E(SCRF) (M06-2X/def2tzvpp): -846,964216 Hartrees
Correction to Enthalpy: 0.405793
Correction to Gibbs Free Energy: 0.334758

Standard orientation:

Center Number	Atomic Number	Atomic Type	Coordinates (Angstroms) X	Y	Z
1	6	0	2.479144	-3.513553	0.300648
2	6	0	3.525840	-2.564607	-0.298413
3	6	0	3.216690	-1.104600	0.060018
4	1	0	3.534895	-2.675961	-1.392214
5	1	0	4.529183	-2.837024	0.047468
6	6	0	1.796482	-0.689530	-0.385082
7	1	0	3.296542	-0.959766	1.146228
8	6	0	0.760232	-1.636632	0.263703
9	6	0	1.053047	-3.102882	-0.096214
10	1	0	0.921662	-3.239990	-1.180049
11	1	0	0.313842	-3.744105	0.395183
12	1	0	2.563153	-3.500959	1.396098
13	1	0	2.669060	-4.546168	-0.013856
14	7	0	-0.617793	-1.294275	-0.070355
15	1	0	0.830508	-1.513756	1.349582
16	1	0	3.946541	-0.426648	-0.398055
17	6	0	-1.351319	-0.428151	0.694565
18	1	0	-0.921371	-1.458052	-1.020092

19	8	0	-0.912777	0.068295	1.733489
20	6	0	-2.740041	-0.126523	0.209049
21	6	0	-3.340846	1.054078	0.664735
22	6	0	-4.625813	1.395713	0.251545
23	6	0	-3.457562	-0.973911	-0.646735
24	6	0	-4.747236	-0.634432	-1.055659
25	6	0	-5.330678	0.553580	-0.611999
26	1	0	-5.079899	2.318183	0.602905
27	1	0	-3.028654	-1.918029	-0.969539
28	1	0	-5.298505	-1.300947	-1.711403
29	1	0	-6.333600	0.818507	-0.932372
30	1	0	1.737146	-0.821643	-1.479734
31	1	0	1.132648	4.085653	1.110886
32	1	0	1.762526	2.591518	1.809541
33	6	0	2.007995	3.419977	1.137672
34	1	0	2.425792	4.855276	-1.301878
35	6	0	2.411761	2.961265	-0.239361
36	6	0	3.080050	3.990666	-1.113935
37	1	0	2.818048	3.991629	1.609738
38	1	0	1.105681	0.861163	0.833666
39	7	0	1.456548	0.695867	-0.101600
40	6	0	2.167262	1.723359	-0.711647
41	1	0	3.361858	3.577098	-2.088192
42	1	0	3.988816	4.391560	-0.643422
43	1	0	2.504138	1.466218	-1.716970
44	1	0	-2.779127	1.685839	1.344913

Complex Enamine(**15**):Maleimide
E(SCRF) (M06-2X/def2tzvpp): -1512.705464 Hartrees
Correction to Enthalpy: 0.573784

Standard orientation:

Center Number	Atomic Number	Atomic Type	Coordinates (Angstroms) X	Y	Z
1	6	0	-1.091052	1.394007	3.945049
2	6	0	-0.587004	3.646996	-0.363951
3	6	0	1.405964	2.135954	-0.651951
4	7	0	1.937935	0.775942	-0.604951
5	1	0	1.205965	2.185958	-2.803951
6	1	0	-0.670968	5.339998	-1.715951
7	1	0	0.367955	1.725976	1.669049
8	1	0	-3.746075	0.293063	3.540049
9	1	0	-0.643047	1.603997	-1.072951
10	1	0	-0.071992	4.212985	0.426049
11	1	0	1.952976	2.702942	0.111049
12	1	0	-3.795038	2.030065	3.851049
13	1	0	1.739022	4.857947	-1.388951
14	1	0	-0.034052	1.368984	3.662049
15	1	0	-1.267070	0.533011	4.601049
16	1	0	2.761977	2.756925	-2.202951
17	1	0	-0.911001	3.784003	-2.497951
18	6	0	3.204930	0.532916	-0.191951
19	6	0	-3.502054	1.274058	3.110049
20	6	0	-2.037052	1.382027	2.770049
21	7	0	-0.348049	1.530991	0.980049
22	6	0	-0.322990	4.300991	-1.726951
23	6	0	-0.097035	2.182986	-0.311951
24	6	0	1.682978	2.791948	-2.020951
25	6	0	1.166009	4.233959	-2.088951

26	6	0	-1.646050	1.494019	1.482049
27	1	0	-4.134051	1.412072	2.228049
28	1	0	-1.655003	3.678019	-0.121951
29	1	0	-2.405050	1.489035	0.702049
30	1	0	1.339018	4.646955	-3.088951
31	1	0	-1.245033	2.289010	4.563049
32	6	0	-0.091115	-1.570014	1.201049
33	6	0	-1.003120	-1.822995	2.147049
34	1	0	1.341919	0.027955	-0.946951
35	6	0	-2.357120	-1.825966	1.527049
36	6	0	3.736900	-0.856096	-0.241951
37	6	0	3.046878	-1.934081	-0.832951
38	6	0	5.019895	-1.101123	0.325049
39	6	0	3.581850	-3.214092	-0.849951
40	1	0	2.076881	-1.773060	-1.290951
41	6	0	5.547868	-2.404134	0.307049
42	6	0	4.837845	-3.444119	-0.270951
43	1	0	3.032833	-4.027081	-1.314951
44	1	0	6.524864	-2.559155	0.752049
45	1	0	5.264824	-4.443128	-0.280951
46	8	0	3.914950	1.470900	0.244049
47	8	0	5.758916	-0.135139	0.886049
48	1	0	5.235934	0.707872	0.760049
49	6	0	-0.792111	-1.382999	-0.101951
50	8	0	-0.290105	-1.130010	-1.190951
51	8	0	-3.425125	-2.034943	2.061049
52	1	0	0.983887	-1.502037	1.282049
53	1	0	-0.863124	-2.014998	3.202049
54	1	0	-4.591092	-0.520919	0.500049
55	1	0	-6.385089	-0.367880	-1.195951
56	6	0	-4.422100	-0.888922	-0.503951
57	6	0	-5.431099	-0.814901	-1.461951
58	6	0	-3.194113	-1.472948	-0.836951
59	6	0	-5.222109	-1.302905	-2.751951
60	1	0	-6.010108	-1.236888	-3.496951
61	6	0	-2.981123	-1.975953	-2.125951
62	6	0	-3.994121	-1.878931	-3.077951
63	1	0	-3.821130	-2.265935	-4.077951
64	7	0	-2.158114	-1.557970	0.142049
65	1	0	-2.029133	-2.422973	-2.380951

Complex Enamine(**19**):Maleimide
E(SCRF) (M06-2X/def2tzvpp): -1437.460085 Hartrees
Correction to Enthalpy: 0.568314

Standard orientation:

Center Number	Atomic Number	Atomic Type	Coordinates (Angstroms) X	Y	Z
1	6	0	-1.000959	1.438060	3.931063
2	6	0	-0.366844	3.651027	-0.386937
3	6	0	1.626077	2.123924	-0.596937
4	7	0	2.148006	0.764897	-0.514937
5	1	0	1.495078	2.153931	-2.754937
6	1	0	-0.399756	5.329029	-1.758937
7	1	0	0.523056	1.727981	1.689063
8	1	0	-3.654015	0.350199	3.462063
9	1	0	-0.411950	1.601030	-1.076937
10	1	0	0.126186	4.222002	0.412063
11	1	0	2.155107	2.695896	0.175063

12	1	0	-3.696925	2.090201	3.763063
13	1	0	1.997218	4.837904	-1.350937
14	1	0	0.062039	1.403005	3.676063
15	1	0	-1.202003	0.585071	4.591063
16	1	0	3.034108	2.722851	-2.106937
17	1	0	-0.621838	3.765041	-2.531937
18	6	0	3.417994	0.533831	-0.070937
19	6	0	-3.389965	1.328185	3.034063
20	6	0	-1.916960	1.423108	2.732063
21	7	0	-0.181953	1.548018	0.985063
22	6	0	-0.055810	4.288011	-1.747937
23	6	0	0.114080	2.184002	-0.305937
24	6	0	1.950110	2.764907	-1.962937
25	6	0	1.443185	4.208933	-2.061937
26	6	0	-1.490954	1.525086	1.454063
27	1	0	-3.997957	1.466216	2.135063
28	1	0	-1.441842	3.690083	-0.179937
29	1	0	-2.229954	1.523124	0.655063
30	1	0	1.650206	4.611923	-3.059937
31	1	0	-1.163912	2.341069	4.537063
32	6	0	0.059887	-1.533995	1.247063
33	6	0	-0.878126	-1.765946	2.173063
34	1	0	1.562967	0.013927	-0.865937
35	6	0	-2.215126	-1.781876	1.518063
36	6	0	3.919920	-0.888196	-0.097937
37	6	0	3.272865	-1.948162	-0.752937
38	6	0	5.130907	-1.135259	0.564063
39	6	0	3.827798	-3.229191	-0.732937
40	1	0	2.340873	-1.793113	-1.284937
41	6	0	5.679841	-2.415287	0.585063
42	6	0	5.028786	-3.466253	-0.063937
43	1	0	3.320756	-4.040164	-1.245937
44	1	0	6.617832	-2.592336	1.105063
45	1	0	5.456734	-4.464276	-0.050937
46	8	0	4.132042	1.443793	0.359063
47	6	0	-0.605105	-1.378960	-0.078937
48	8	0	-0.074094	-1.156988	-1.159937
49	8	0	-3.297137	-1.980820	2.028063
50	1	0	1.131890	-1.461050	1.354063
51	1	0	-0.766134	-1.931952	3.236063
52	1	0	-4.420060	-0.502762	0.408063
53	1	0	-6.169054	-0.383671	-1.336937
54	6	0	-4.225080	-0.890772	-0.583937
55	6	0	-5.209077	-0.835721	-1.569937
56	6	0	-2.988111	-1.479836	-0.872937
57	6	0	-4.967104	-1.348733	-2.843937
58	1	0	-5.735101	-1.297693	-3.609937
59	6	0	-2.742138	-2.006849	-2.146937
60	6	0	-3.731134	-1.930797	-3.126937
61	1	0	-3.531155	-2.335808	-4.113937
62	7	0	-1.979114	-1.543889	0.134063
63	1	0	-1.784162	-2.457899	-2.367937
64	1	0	5.622951	-0.300284	1.049063

TS-1R

E(SCRF) (M06-2X/def2tzvpp): -1512,6899 Hatrees

Correction to Enthalpy: 0.573192

Correction to Gibbs Free Energy: 0.480294

Standard orientation:

Center Number	Atomic Number	Atomic Type	Coordinates (Angstroms) X	Y	Z
1	6	0	-0.165957	-2.983569	-2.536312
2	6	0	1.329964	2.057505	-2.336587
3	6	0	-0.888016	1.810768	-1.135496
4	7	0	-1.659990	0.967982	-0.225276
5	1	0	-0.329020	3.287144	0.340129
6	1	0	1.974927	4.103446	-2.616105
7	1	0	-0.507981	-0.605316	-1.498900
8	1	0	2.301063	-4.241536	-2.564031
9	1	0	1.036004	1.230992	-0.361377
10	1	0	0.842952	2.002252	-3.318567
11	1	0	-1.418028	1.794521	-2.093485
12	1	0	2.288027	-3.093870	-3.905317
13	1	0	-0.502074	4.198412	-2.589096
14	1	0	-0.989956	-2.553439	-1.961408
15	1	0	-0.188937	-4.064528	-2.370042
16	1	0	-1.803038	3.665898	-0.551946
17	1	0	2.007958	3.563863	-0.941971
18	6	0	-3.010990	0.841923	-0.374227
19	6	0	2.350043	-3.177598	-2.814297
20	6	0	1.203040	-2.425450	-2.146469
21	7	0	0.386009	-0.190363	-1.746015
22	6	0	1.422947	3.519622	-1.870952
23	6	0	0.522991	1.220742	-1.333368
24	6	0	-0.788033	3.269889	-0.656861
25	6	0	0.032940	4.124659	-1.632084
26	6	0	1.351016	-1.000446	-2.140826
27	1	0	3.324041	-2.811499	-2.481401
28	1	0	2.337969	1.645492	-2.452498
29	1	0	2.334008	-0.559464	-2.274948
30	1	0	0.125929	5.146756	-1.248340
31	1	0	-0.361973	-2.805837	-3.599354
32	6	0	0.238069	-2.151819	0.449476
33	6	0	1.278070	-2.779988	-0.295382
34	1	0	-1.192974	0.663203	0.632794
35	6	0	2.567065	-2.126849	0.178439
36	6	0	-3.782964	0.071161	0.629975
37	6	0	-3.199940	-0.516546	1.771114
38	6	0	-5.183965	-0.078913	0.425031
39	6	0	-3.964916	-1.231332	2.681302
40	1	0	-2.133939	-0.419483	1.956076
41	6	0	-5.946941	-0.805694	1.356222
42	6	0	-5.342917	-1.372407	2.467355
43	1	0	-3.494898	-1.674107	3.553406
44	1	0	-7.010941	-0.901757	1.172260
45	1	0	-5.947898	-1.930240	3.177502
46	8	0	-3.575012	1.356666	-1.368348
47	8	0	-5.823987	0.444814	-0.630091
48	1	0	-5.126002	0.918693	-1.161218
49	6	0	0.746060	-1.016644	1.120186
50	8	0	0.176053	-0.106496	1.750967
51	8	0	3.707068	-2.477893	-0.072489
52	1	0	-0.800925	-2.442823	0.502562
53	1	0	1.336086	-3.863013	-0.401113
54	1	0	4.487029	-0.164895	-0.133075
55	1	0	6.055016	1.463377	0.859499
56	6	0	4.261035	0.287339	0.822815
57	6	0	5.148027	1.197496	1.395577
58	6	0	3.092050	-0.076509	1.507921

59	6	0	4.878035	1.762802	2.642440
60	1	0	5.571029	2.473923	3.083254
61	6	0	2.824057	0.480800	2.766786
62	6	0	3.714049	1.400952	3.319545
63	1	0	3.494055	1.829191	4.292442
64	7	0	2.187057	-1.007670	0.922165
65	1	0	1.919068	0.206915	3.289866

TS-1S

E(SCRF) (M06-2X/def2tzvpp): -1512,67939 Hartrees

Correction to Enthalpy: 0.572736

Correction to Gibbs Free Energy: 0.477886

Standard orientation:

Center Number	Atomic Number	Atomic Type	Coordinates (Angstroms) X	Y	Z
1	1	0	0.003978	-3.610992	-2.914984
2	1	0	-1.688026	-3.212008	-2.506984
3	6	0	-0.723023	-3.520999	-2.099984
4	1	0	-0.861014	-4.518000	-1.671984
5	1	0	-1.645072	1.569992	0.311016
6	1	0	3.541955	-1.204958	1.655016
7	6	0	-0.220033	-2.553994	-1.028984
8	8	0	2.428944	-0.066969	1.186016
9	7	0	0.199945	-0.199990	-0.670984
10	8	0	4.452958	-1.583949	1.555016
11	1	0	1.882972	-3.059974	-1.196984
12	1	0	0.855926	1.812016	0.947016
13	6	0	-1.362074	1.762995	-0.729984
14	1	0	-0.743095	3.957001	0.892016
15	6	0	1.106972	-3.023981	-0.422984
16	1	0	0.994982	-4.034982	-0.018984
17	1	0	-2.065068	1.202988	-1.354984
18	6	0	-0.288046	-1.174995	-1.417984
19	1	0	-2.482091	3.604984	-0.834984
20	6	0	0.065931	1.240009	-0.965984
21	6	0	-1.459088	3.264994	-1.024984
22	6	0	1.080924	2.024018	-0.103984
23	6	0	-0.465096	4.053004	-0.164984
24	6	0	3.091937	0.640038	0.386016
25	6	0	5.137950	-0.723943	0.789016
26	1	0	1.466966	-2.383978	0.384016
27	7	0	2.466928	1.604032	-0.334984
28	6	0	0.965909	3.536017	-0.360984
29	1	0	-0.902049	-0.872000	-2.262984
30	1	0	-0.503106	5.122003	-0.404984
31	6	0	4.542939	0.423052	0.195016
32	1	0	6.924961	-1.874926	1.029016
33	1	0	1.664904	4.057024	0.302016
34	6	0	6.502953	-0.985930	0.573016
35	1	0	0.333930	1.378011	-2.023984
36	1	0	-1.257090	3.454996	-2.088984
37	1	0	2.989923	2.100037	-1.039984
38	1	0	1.287907	3.745020	-1.392984
39	6	0	5.361931	1.293059	-0.552984
40	6	0	7.272944	-0.120922	-0.185984
41	6	0	6.708933	1.035072	-0.747984
42	1	0	4.947922	2.208055	-0.968984
43	1	0	8.326946	-0.334912	-0.334984

44	1	0	7.319927	1.722078	-1.322984
45	8	0	-1.981060	0.278989	2.343016
46	1	0	-4.381050	-0.687034	-1.662984
47	6	0	-1.025040	-1.782002	1.508016
48	6	0	-4.784056	-0.115038	-0.838984
49	1	0	-6.401064	0.692947	-1.992984
50	6	0	-5.932063	0.655951	-1.013984
51	6	0	-1.366032	-2.638005	0.421016
52	7	0	-2.998048	-0.969020	0.604016
53	6	0	-4.170055	-0.187032	0.423016
54	6	0	-2.732036	-2.160018	-0.057984
55	6	0	-6.479070	1.370946	0.052016
56	6	0	-4.724062	0.524963	1.499016
57	1	0	-7.373076	1.970938	-0.088984
58	6	0	-5.867069	1.299952	1.304016
59	8	0	-3.444031	-2.712025	-0.884984
60	1	0	-4.245061	0.479968	2.467016
61	1	0	-6.283075	1.845948	2.146016
62	6	0	-1.952050	-0.712011	1.614016
63	1	0	-0.204039	-1.895994	2.202016
64	1	0	-1.306021	-3.723004	0.522016
65	1	0	0.717947	-0.435985	0.177016

TS-2R

E(SCRF) (M06-2X/def2tzvpp): -1437,436523 Hartrees

Correction to Enthalpy: 0.567603

Correction to Gibbs Free Energy: 0.476041

Standard orientation:

Center Number	Atomic Number	Atomic Type	Coordinates (Angstroms) X	Y	Z
1	1	0	-0.902025	4.444003	2.348029
2	1	0	-2.378020	3.604995	1.801029
3	6	0	-1.424023	4.026000	1.479029
4	1	0	-1.652027	4.854999	0.802029
5	1	0	-1.728995	-0.887001	0.944029
6	6	0	-0.550017	2.992005	0.770029
7	8	0	2.562996	0.787023	-0.626971
8	7	0	0.327996	0.764010	1.053029
9	1	0	1.367978	3.920016	1.178029
10	1	0	0.685007	-1.212988	-0.543971
11	6	0	-1.093994	-1.149998	1.793029
12	1	0	-1.114982	-3.145998	-0.094971
13	6	0	0.778980	3.599013	0.310029
14	1	0	0.587975	4.485012	-0.303971
15	1	0	-1.495996	-0.631000	2.670029
16	6	0	-0.483010	1.726006	1.448029
17	1	0	-2.215984	-2.949004	2.150029
18	6	0	0.335004	-0.645990	1.513029
19	6	0	-1.167985	-2.664998	2.011029
20	6	0	1.086008	-1.484986	0.440029
21	6	0	-0.559981	-3.401995	0.816029
22	6	0	3.171001	-0.198974	-0.186971
23	6	0	5.379995	0.899038	-0.445971
24	1	0	1.389984	2.913016	-0.279971
25	7	0	2.538007	-1.223977	0.443029
26	6	0	0.906017	-2.997987	0.649029
27	1	0	-1.252008	1.465001	2.168029
28	1	0	-0.629975	-4.486995	0.950029

29	6	0	4.663002	-0.295966	-0.300971
30	1	0	7.316990	1.808049	-0.658971
31	1	0	1.363020	-3.514984	-0.201971
32	6	0	6.767995	0.877046	-0.554971
33	1	0	0.924004	-0.698986	2.442029
34	1	0	-0.637984	-2.946995	2.932029
35	1	0	3.115011	-1.923974	0.884029
36	1	0	1.464018	-3.304983	1.549029
37	6	0	5.354008	-1.515962	-0.296971
38	6	0	7.453002	-0.339950	-0.535971
39	6	0	6.744009	-1.536954	-0.412971
40	1	0	4.813014	-2.455965	-0.243971
41	1	0	8.535002	-0.357944	-0.624971
42	1	0	7.270014	-2.485951	-0.419971
43	8	0	-1.131995	-0.828998	-1.930971
44	1	0	-4.514003	0.604983	1.075029
45	6	0	-0.679009	1.526005	-1.580971
46	6	0	-4.645999	-0.194018	0.358029
47	1	0	-6.382994	-0.989027	1.333029
48	6	0	-5.704994	-1.090024	0.489029
49	6	0	-1.356014	2.539001	-0.836971
50	7	0	-2.685003	0.610993	-0.869971
51	6	0	-3.765998	-0.299013	-0.731971
52	6	0	-2.725011	1.953993	-0.512971
53	6	0	-5.898988	-2.105025	-0.447971
54	6	0	-3.964993	-1.315014	-1.680971
55	1	0	-6.724984	-2.802029	-0.339971
56	6	0	-5.021988	-2.210020	-1.527971
57	8	0	-3.671014	2.545988	-0.014971
58	1	0	-3.280992	-1.403010	-2.513971
59	1	0	-5.162983	-2.992020	-2.268971
60	6	0	-1.405002	0.312001	-1.541971
61	1	0	0.275991	1.606010	-2.078971
62	1	0	-1.410020	3.565001	-1.204971
63	1	0	1.047995	0.976014	0.347029
64	1	0	4.828990	1.833035	-0.471971

TS-2S

E(SCRF) (M06-2X/def2tzvpp): -1437,444912 Hatrees

Correction to Enthalpy: 0.567739

Correction to Gibbs Free Energy: 0.476092

Standard orientation:

Center Number	Atomic Number	Atomic Type	Coordinates (Angstroms) X	Y	Z
1	6	0	-0.332924	-3.047025	-2.471021
2	6	0	1.235013	1.975995	-2.394021
3	6	0	-1.019985	1.797966	-1.249021
4	7	0	-1.829975	0.994956	-0.340021
5	1	0	-0.485004	3.319973	0.189979
6	1	0	1.913987	4.003003	-2.724021
7	1	0	-0.668954	-0.626029	-1.514021
8	1	0	2.117093	-4.334994	-2.409021
9	1	0	0.873022	1.221990	-0.401021
10	1	0	0.776014	1.892989	-3.387021
11	1	0	-1.524984	1.756960	-2.221021
12	1	0	2.150079	-3.228994	-3.784021
13	1	0	-0.561014	4.128972	-2.773021
14	1	0	-1.161929	-2.587036	-1.926021

15	1	0	-0.374910	-4.122026	-2.271021
16	1	0	-1.928009	3.682955	-0.759021
17	1	0	1.891993	3.522003	-1.033021
18	6	0	-3.187974	0.897939	-0.534021
19	6	0	2.186079	-3.279993	-2.689021
20	6	0	1.034069	-2.494008	-2.071021
21	7	0	0.240041	-0.237018	-1.754021
22	6	0	1.333994	3.452996	-1.976021
23	6	0	0.388023	1.185984	-1.387021
24	6	0	-0.915004	3.272967	-0.819021
25	6	0	-0.055014	4.082978	-1.799021
26	6	0	1.201051	-1.072006	-2.101021
27	1	0	3.156075	-2.915981	-2.345021
28	1	0	2.241018	1.548007	-2.466021
29	1	0	2.193046	-0.648993	-2.226021
30	1	0	0.040973	5.115980	-1.449021
31	1	0	-0.506926	-2.900027	-3.543021
32	6	0	0.015065	-2.143021	0.499979
33	6	0	1.066073	-2.797008	-0.202021
34	1	0	-1.402971	0.730961	0.549979
35	6	0	2.348065	-2.136991	0.279979
36	6	0	-3.989964	0.166929	0.508979
37	6	0	-3.445957	-0.403065	1.669979
38	6	0	-5.368963	0.064911	0.274979
39	6	0	-4.277949	-1.063075	2.576979
40	1	0	-2.382958	-0.346051	1.886979
41	6	0	-6.193955	-0.594099	1.181979
42	6	0	-5.648948	-1.160092	2.336979
43	1	0	-3.848943	-1.500070	3.473979
44	1	0	-7.259954	-0.666113	0.989979
45	1	0	-6.290941	-1.675101	3.046979
46	8	0	-3.725979	1.368932	-1.537021
47	6	0	0.514050	-0.992015	1.152979
48	8	0	-0.063961	-0.066022	1.750979
49	8	0	3.492069	-2.501977	0.062979
50	1	0	-1.027932	-2.424034	0.533979
51	1	0	1.120087	-3.882007	-0.280021
52	1	0	4.293040	-0.204967	-0.037021
53	1	0	5.849020	1.441053	0.945979
54	6	0	4.048034	0.278030	0.899979
55	6	0	4.928023	1.199041	1.466979
56	6	0	2.859039	-0.055985	1.565979
57	6	0	4.632015	1.804038	2.688979
58	1	0	5.319006	2.523046	3.124979
59	6	0	2.565031	0.541011	2.799979
60	6	0	3.448019	1.470023	3.346979
61	1	0	3.208013	1.930020	4.301979
62	7	0	1.961050	-0.997996	0.986979
63	1	0	1.644034	0.290000	3.308979
64	1	0	-5.765969	0.512906	-0.628021
